# A Strategy for International Cooperation in the COVID-19 Pandemic Era: Focusing on National Scientific Funding Data

**DOI:** 10.3390/healthcare8030204

**Published:** 2020-07-09

**Authors:** Doyeon Lee, Yoseob Heo, Keunhwan Kim

**Affiliations:** 1 Division of Data Analysis, Korea Institute of Science and Technology Information (KISTI), Seoul 02456, Korea; dylee@kisti.re.kr; 2 Busan Branch, Division of Data Analysis, Korea Institute of Science and Technology Information (KISTI), Busan 48058, Korea; joseph87@kisti.re.kr

**Keywords:** coronavirus, international cooperation, national scientific funds, cluster analysis, pandemic, health policy

## Abstract

The coronavirus crisis may lead to a deeper understanding of international collaborations for developing antivirals and vaccines that are essential to protect us from current and future health security threats. Beyond technical solutions, the government of South Korea needs to establish a timely strategic investment in coronavirus-related research and development (R&D) in order to enhance the capabilities for managing this new uncertainty in regard to the domestic health crisis. Thus, this study aims to provide useful information about the status of global coronavirus-related research from the South Korean government’s perspective. National funded projects stemmed from leading nations such as the United States, countries of the European Union, and Japan between 2012 and 2018. Six research fields were derived by clustering analysis and an expert-based approach, and then matched to those of South Korea. The comparative analysis among them allowed for the identification of the nations’ strengths and weaknesses, thereby laying the groundwork for strategic international research collaborations.

## 1. Introduction

The third zoonotic human coronavirus of the century emerged in December 2019, with a cluster of patients with connections to Huanan South China Seafood Market in Wuhan, Hubei Province, China [1,2]. The cases of those infected in China had spread to the US, Thailand, Japan, and South Korea (hereafter Korea) on a smaller scale by early February. The confirmed cases in other countries increased [3], and a global pandemic of COVID-19 caused by severe acute respiratory syndrome coronavirus 2 (SARS-CoV-2) was eventually declared by the World Health Organization on 11 March 2020 [4].

The world has already learned the importance of working collaboratively to overcome two global outbreaks, which stemmed from the SARS outbreak of 2002 and the MERS outbreak of 2012. After the SARS outbreak in 2003, it took researchers about 20 months to develop a vaccine for human trials. In 2015, researchers were able to prepare a vaccine for the Zika virus in just six months [5].

As the world is increasingly interconnected and the COVID-19 epidemic continues [6], it is of the utmost importance for both the developed and developing nations to facilitate international cooperation and to protect the health of the world’s population [7,8,9]. At the center of the WHO, its networks of researchers and other experts have coordinated global work on surveillance, epidemiology, modeling, diagnostics, clinical care and treatment, and on researching other ways to identify and manage the disease and limit its onward transmission [5,9], thereby reducing the probability of the high lethality of viruses such as SARS-CoV, MERS-CoV, H5N1, H7N9, and Ebola [10]. In order to contribute to the treatment and prevention of the virus with the collaborative efforts of countries around the world, much research will continue to be conducted in the fields of pathogen monitoring, source identification, primary etiology, clinical treatments, vaccine development, viral replication, pathogenesis, antiviral drugs, and others [9,10,11]. The US CDC, as well as other national governmental bodies, has the explicit goal of preventing sustained outbreaks like COVID-19 within their borders but also makes significant contributions to ongoing publicly shared research in disease transmission, diagnostics, vaccine development, and therapeutics, as well as offering guidelines to front-line clinicians and coordinating with both the WHO and local health departments [12].

Although the world is already preparing to tackle the newest emergent virus strain [1], vaccine research and development are highly complex and require concerted public funding efforts for a long period of time [8,13]. In order to improve the efficiency of the allocation of the funding for infectious disease-related R&D, it is necessary to understand the current trends of coronavirus disease-related scientific research funding around the world to consider the directions for the global collaborative research and development [14,15].

It was first reported that a patient in Korea was infected with COVID-19 on 20 January 2020 in Korea (ncov.hohw.go.kr); community-based spread dramatically occurred in Korea [16], thereby positioning it as one of the first countries to have an outbreak beside China, based on reported laboratory-confirmed COVID-19 cases. As of 6 March 2020, 17,481 cases of COVID-19 had been confirmed outside China, 6284 of which were confined to South Korea (42 deaths). Italy (3858 confirmed cases and 148 deaths) and Iran (3513 confirmed cases and 107 deaths) were ranked next (WHO 2020 Situation Report-46) [3].

The Korean government and its other agencies, such as the Korea Center for Disease Control (KCDC), have made saving lives their highest priority by implementing comprehensive testing and tracking, which allowed for the early detection, isolation, and treatment of patients while minimizing widespread mobility restrictions. As a result of the cooperation between government agencies and the wider public, the number of confirmed cases slowed down to an average of less than 10 per day in May, compared with more than 500 new cases per day in late February and early March. During the COVID-19 crisis, the Korean government’s rapid response to the epidemic resulted in the substantial improvement of the national scientific research ability and research equipment through the integration of scientific research resources, increase in research investment, strengthening of direct cooperation between international and domestic scientists, and acceleration of the clinical applications of scientific research results [17], thereby enhancing the ability to prevent the spread of the epidemic or accelerate the elimination of the virus [18]. The Korean government was able to pause and reflect after the peak of the coronavirus crisis started to subside.

Due to the magnitude of the public funds invested in infectious disease-related research [14], the Korean government has to evaluate their investment strategy about which research fields must be invested in and in which research fields is needed collaboration with international partners to reach important research goals [13]. To date, few studies have provided a comprehensive analysis of infectious disease R&D from a public funding perspective [13,14,15]. To the best of our knowledge, there is no study to satisfy both prerequisites for understanding what coronavirus disease-related research and development has been conducted from both the world’s and South Korea’s perspectives. Therefore, this study aims to provide useful information about the coronavirus disease-related scientific research trends of leading nations and Korea during the 2012–2018 time period and then analyze this research to plan further scientific research. Many studies have indicated that the US, EU, and Japan have critical roles in the scientific and technological advancements in infectious diseases [14,19,20]. In particular, as the four leading national scientific funding organizations or programs related to the health domain in the world, the National Institutes of Health (NIH) of the US; the framework programs for research and innovation (i.e., Horizon 2020) funded by the EU; and the Grants-in-Aid for Scientific Research (KAKENHI) program of the Ministry of Education, Science and Culture of the Japanese government are significant [21,22]. Therefore, the data from government-funded projects were gathered from searchable, open, project-based information databases such as STAR METRICS, CORDIS (Community Research & Development Information Service, Brussels, Belgium), and KAKEN (Database of Grants-in-Aid for Scientific Research, Shizuoka, Japan), respectively, that are available for individual or multiple institutions in those places. Besides, the official centralized national R&D database called the National Science & Technology Information Service (NTIS)—which has gathered, managed, and provided all Korean government-funded research information since 2004 (www.ntis.go.kr)—was utilized. This paper aims to apply machine learning methodologies and network analysis to understand how the US, EU, and Japan have invested their funding to determine what coronavirus disease-related research fields already exist and match those to the Korean projects with designated research fields stemming from leading nations’ data analysis. Our research addresses the following questions:

1. What coronavirus-related research has been conducted amongst leading nations since 2012?
2. What coronavirus-related R&D fields has Korea invested in since 2012?
3. What differences exist amongst coronavirus-related R&D fields?
4. What insights into coronavirus-related R&D fields has Korea gained in order to prepare for the post-COVID-19 pandemic?

The remainder of this paper consists of four sections. Following this general introduction, the “materials and methods” section describes the framework and methodology. The “results” section presents comparative results of the research profiling and machine learning analyses. The “conclusion and discussion” section reviews our research, identifies the research limitations, and indicates promising research opportunities to pursue.

## 2. Materials and Methods

### 2.1. Data Collection

The data used in this study are national-funded R&D project information and were collected from the global R&D database provided by STAR METRICS of the US, CORDIS of the EU, and KAKEN of Japan. The global R&D database was built and is operated by the Korea Institute of Science and Technology Information (KISTI) internally, funded by the Ministry of Science and ICT of Korea. It has data from approximately 1 million nationally funded projects between 2012 and 2018. The detailed process of database establishment is described in [15]. The data from Korea were collected from NTIS, which is utilized in many studies of Korean R&D trends [23,24,25]. A total of 599 and 638 nationally funded R&D projects related to coronaviruses between 2012 and 2018 were collected from the developed nations (the US, EU countries, and Japan) and Korea with these query sets in Table 1, respectively.

### 2.2. Data Pre-Processing for Further Analysis

None of the national R&D databases provide the complete information of all nationally funded projects. For example, suppose that a five-year nationally funded scientific project was started in June 2015. STAR-METRICS and NTIS contained funding information for some fiscal years, which did not allow us to estimate the total amount of funds for multi-year projects. Thus, calculations were estimated by multiplying the average funding amount per year that was extrapolated based on the title of these projects in the global R&D database and the total period (year) of these projects. After that, we removed some data that included the number of funded projects that were zero and kept some data by adding the organization names as found through manual searches for these projects from other data sources. Table 1 shows a total of 273 and 170 nationally funded R&D projects related to coronaviruses utilized for further analysis, respectively.

### 2.3. Clustering Through Co-Occurrence Matrix

As a way of identifying coronavirus-related R&D areas, the co-occurrence matrix was made in terms of the ASJC code (All Science Journal Classification Codes) of Scopus by using the Vantage Point^®^ system (Search Technology, Inc., Norcross, GA, USA) as demonstrated in [15]. From the single standard’s perspective, the ASJC code was previously assigned to all the projects in the global R&D database that employed the machine learning approach to classify the different R&D projects that stemmed from the US, the EU, and Japan [15]. The number of data utilized by Korea, which has the characteristics of the centralized database, was as high as that used by the US. It may cause a “home advantage” bias [26,27]. Thus, the data of Korea excluded the making of a co-occurrence matrix.

Identifying the association among ASJC codes through the co-occurrence matrix implied a network structure. Thus, the VOSViewer (Leiden University, Gravenhage, the Netherlands) software was used as a network structure visualization tool to understand the relationship between ASJC codes [15]. The VOSViewer system calculates the similarity between each component and visualizes the network structure in the form of a cluster map or a topographic map [28]. A mathematical model and algorithm of VOSviewer’s clustering and mapping can be found in Van Eck and Waltman [29]. The software has been used in various studies to identify the research fields [30,31].

### 2.4. Sub-Clustering and Definitions of (Sub-)Clusters

The constructed clusters were initially subdivided into more detailed sub-clusters. Therefore, in order to derive more coronavirus-related R&D areas from more massive clusters, each larger cluster was divided into several sub-clusters. After that, the definitions of coronavirus-related R&D areas were determined by directly and carefully reviewing R&D projects comprised of clusters or sub-clusters.

### 2.5. Allocating All the Projects to (Sub-)Clusters

**Figure 1:**
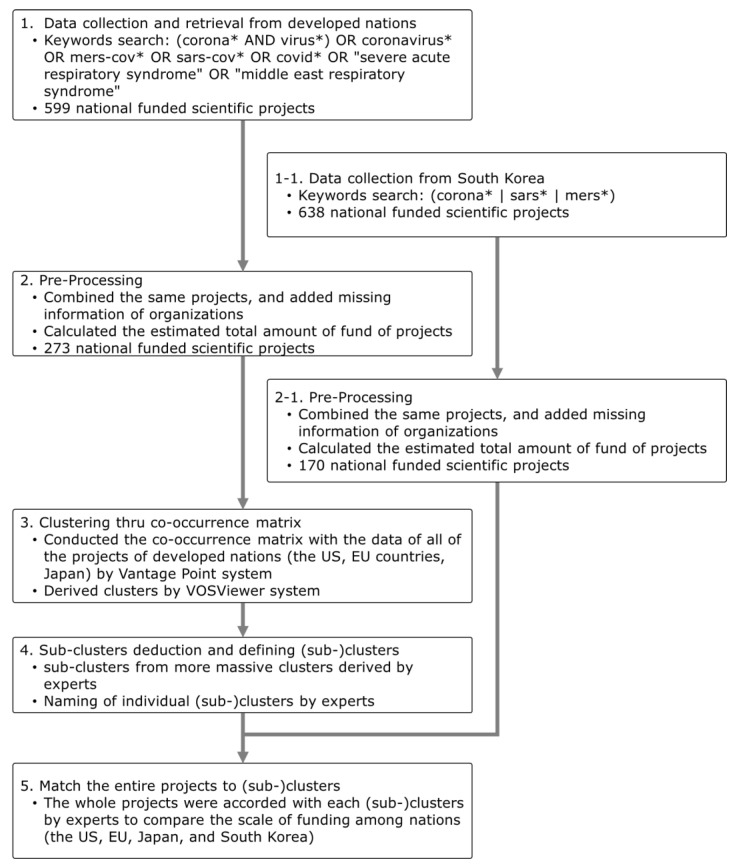
The workflow of the comparison of coronavirus-related R&D areas among the US, the EU, Japan, and South Korea. Search terms marked with an asterisk (*) are root or stem words, indicating that all possible suffixes are covered under the query.

The clustering techniques drop many data from the original data [15], which prevents us from comparing the amount of R&D funding among the US, the EU, Japan, and Korea. Thus, all of the projects that were excluded from each (sub-)cluster were accorded with each (sub-)cluster or removed if they did not belong to any cluster according to experts. We sought to draw out the significant implications for the directions of the coronavirus-related R&D planning of Korea through the comparisons between the nations. The whole process is shown in Figure 1.

## 3. Results

### 3.1. Research and Development Fields of Coronavirus-Related National Funded Projects of the US, the EU, and Japan

**Figure 2:**
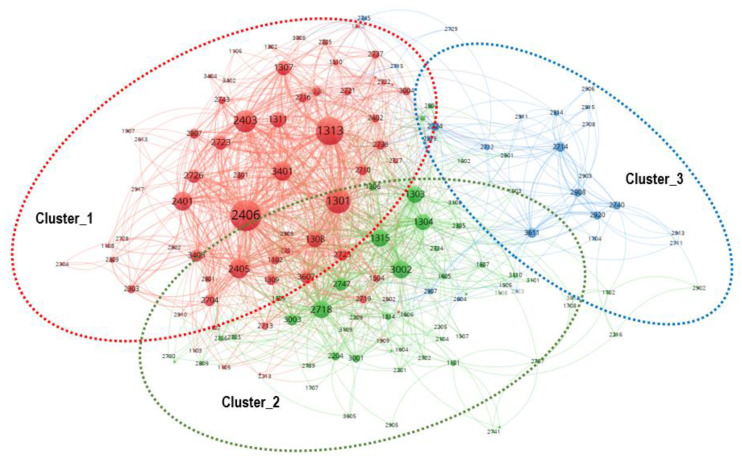
Clusters of R&D fields on coronavirus-related national-funded projects of the US, the EU, and Japan.

As shown in Figure 2, the scientific research fields of the coronavirus-related national funded projects in the US, the EU, and Japan may be divided into three clusters (categories). After reviewing the research descriptions of the funded projects, distinct subjects in significant clusters such as Cluster 1 and 2 were deduced by experts and named to include the core meanings of the individual research fields as follows: (Cluster 1) research on the molecular characteristics of infectious viruses and the interaction mechanisms of viral pathogens and human hosts, including the immune response, for developing diagnostics, therapeutics, and a vaccine against COVID-19; (Sub-Cluster 1-1) research related to the mechanisms of infection, the life cycle of SARS-CoV-2, and the identification of a virus–host interaction mechanism; (Sub-Cluster 1-2) research related to the platform for the immunological response to viral infection and for vaccine development; (Sub-Cluster 1-3) the platform for detection and point-of-care diagnostics; (Cluster 2) research on the virus protein structure-active/function-based antivirus therapeutics (treatment) design and active/resistance modulation research on the virus protein structure-active/function-based antivirus therapeutics (treatment) design and active/resistance modulation; (Sub-Cluster 2-1) structure–activity relationship modeling-based virus prediction and activity modulation; (Sub-Cluster 2-2) studies on the design of antiviral agents based on the structure and function of viral and human receptor proteins; and (Cluster 3) infectious disease epidemiological investigation and animal and environmental ecology (all of the national funded projects in each (sub-)cluster are provided in the supplementary material). The next sub-sector describes the detailed investigation for each cluster.

#### 3.1.1. Research on the Molecular Characteristics of Infectious Viruses and the Interaction Mechanism of Viral Pathogens and Human Hosts, Including the Immune Response, for Developing Diagnostics, Therapeutics, and a Vaccine against COVID-19 (Cluster 1)

Research on the molecular characteristics of infectious viruses and the interaction mechanism of viral pathogens and human hosts, including the immune response, for developing diagnostics, therapeutics, and a vaccine against COVID-19 (Cluster 1) were studied in 135 projects, which totaled 367,501,552 USD.

Firstly, research related to the mechanisms of infection, the life cycle of SARS-CoV-2, and the identification of a virus–host interaction mechanism (Sub-Cluster 1-1) in general consisted of 81 projects worth 186,810,747 USD. The University of Maryland in the US recently completed a project called “Host, pathogen, and the microbiome: determinants of infectious disease outcomes” and spent 19.6 million USD between 2014 and 2019. The University of Pittsburgh and the University of Iowa have participated in projects entitled “Targeting host responses to prevent virus induced ARDS in the nonhuman primate model” and “Role of eicosanoids in pathogenic human CoV infections”, respectively. Meanwhile, Erasmus Universitair Medisch Centrum Rotterdam of the EU and Yamaguchi University of Japan conducted a project entitled “European management platform for emerging and reemerging infectious disease entities” with expenditures of 18.6 million USD between 2009 and 2014, and “Elucidation of the mutation mechanism and virulence acquisition machine of coronavirus” with a cost of 514,800 USD between 2015 and 2018, respectively (see Table 2).

Secondly, research related to the platform for an immunological response to viral infection and vaccine development (Sub-Cluster 1-2) was comprised of 38 projects worth 146,167,160 USD. The University of North Carolina Chapel Hill in the US is projected to have spent 24 million USD on its “Systems immunogenetics of biodefense and emerging pathogens in the collaborative cross” by the time the 10 year project wraps up in 2022. The New York Blood Center committed to spending 4.5 million USD on a vaccine project titled “Structure-based design of coronavirus subunit vaccines” between 2018 and 2023. Meanwhile, The National Institute of Infectious Diseases of Japan finished a vaccine project costing 144,300 USD between 2016 and 2019 (see Table 3).

Thirdly, the platform for the detection and point-of-care diagnostics (Sub-Cluster 1-3) is comprised of 16 projects, which are worth 34,526,645 USD. In the US, Columbia University Health Sciences completed a 5 million USD project entitled “Diagnostic and prognostic biomarkers for severe viral lung disease” between 2014 and 2019. Meanwhile, Erasmus Universitair Medisch Centrum Rotterdam in the EU worked on a study between 2014 and 2016 entitled “Early detection of emerging viruses by next generation in situ hybridization” with a budget of 205,891 USD. Between 2015 and 2018, Nihon University of Japan completed its study “Establishment of gene diagnosis of feline infectious peritonitis by the analysis of the viral genome” with expenditures of 132,600 USD (see Table 4).

#### 3.1.2. Research on the Virus Protein Structure, Activity/Function-Based Antivirus Therapeutics (Treatment) Design, and Activity/Resistance Modulation (Cluster 2)

Overall, research on the virus protein structure, activity/function-based antivirus therapeutics (treatment) design, and activity/resistance modulation (Cluster 2) was comprised of 37 projects worth 83,537,310 USD.

First, structure–activity relationship modeling-based virus prediction and activity modulation (Sub-Cluster 2-1) included six projects totaling 6,911,261 USD. In the US, The Blood Systems Research Institute joined a 2 million USD project, “Modeling viral entry and its inhibition using SARS-CoV”, between 2007 and 2012. Harvard University worked on a study between 2013 and 2016 entitled “Structure and mechanism of programmed ribosomal frameshifting in SARS coronavirus”. In the EU, Katholieke Universiteit Leuven completed a 3.5 million USD project, entitled “Virus discovery and epidemic tracing from high throughput metagenomic sequencing”, between 2015 and 2018. Meanwhile, Osaka University of Japan studied the same research area with expenditures of 144,300 USD between 2016 and 2019 (See Table 5).

Secondly, studies on the design of antiviral agents based on the structure and function of viral and human receptor proteins (Sub-Cluster 2-2) included 31 projects totaling 76,626,049 USD. In the US, The University of North Carolina Chapel Hill and Kansas State University committed to spending 6.5 million USD on a project named “Broad-spectrum antiviral GS-5734 to treat MERS-CoV and related emerging CoV” between 2017 and 2022, and to 3.8 million USD worth of a project entitled “Small molecule protease inhibitors against MERS-CoV” between 2018 and 2023. In Japan, the Nippon Veterinary and Life Science University finished “The development of specific anticoronavirus drugs using the novel glycosidase inhibitors”, spending 144,000 USD between 2016 and 2019 (see Table 6).

#### 3.1.3. Infectious Disease Epidemiological Investigation and Animal and Environmental Ecology (Cluster 3)

Infectious disease epidemiological investigation and animal and environmental ecology (Cluster 3) contained 25 projects worth 152,373,487 USD. In 2018, George Washington University in the US began executing a project worth 9.9 million USD titled “Ecology of MERS-CoV in camels, humans, and wildlife in Ethiopia”. It will be completed in 2022. In the EU, Erasmus Universitair Medisch Centrum Rotterdam committed to an 18 million USD project entitled “Anticipating the global onset of novel epidemics” between 2018 and 2023. In Japan, the National Institute of Infectious Diseases recently completed “The survey of middle east respiratory syndrome coronavirus of the dromedary in Ethiopia” project with total expenditures of 413,400 USD between 2017 and 2020 (see Table 7).

### 3.2. Research and Development Fields of Coronavirus-Related National Funded Projects of Korea

#### 3.2.1. Research on the Molecular Characteristics of Infectious Viruses and the Interaction Mechanisms of Viral Pathogens and Human Hosts, Including the Immune Response, for Developing Diagnostics, Therapeutics, and a Vaccine against COVID-19 in Korea (Cluster 1)

Overall, research on the molecular characteristics of infectious viruses and the interaction mechanisms of viral pathogens and human hosts, including immune responses, for developing diagnostics, therapeutics, and a vaccines against COVID-19 in Korea (Cluster 1) was comprised of 121 projects worth 64,518,268 USD.

Firstly, research related to the mechanisms of infection, the life cycle of SARS-CoV-2, and the identification of a virus–host interaction mechanism in Korea (Sub-Cluster 1-1) consisted of 34 projects, totaling 4,351,252 USD. Kookmin University’s project, “Reaction mechanism study on SARS Coronavirus helicase and its applications to develop the inhibitors” spent 137,209 USD between 2013 and 2016. Chonbuk National University and Chungnam National University have participated in the same research areas. Chonbuk National University’s “A genetic characteristic study for zoonotic potential of several pathogens from bats in Korea” has expected expenditures 45,455 USD. The study began in 2018 and will end in 2021. Chungnam National University’s project, “Identification of the mechanism of coronavirus cross-species transmission and pathogenesis mediated by host proteases” has expected expenditures of 136,364 USD between 2020 and 2023 (see Table 8).

Secondly, research related to the platform for the immunological response to viral infection and for vaccine development in Korea (Sub-Cluster 1-2) is composed of 39 projects worth 37,045,703 USD. Seoul National University has concentrated their project on “The development of therapeutic antibodies against Middle Eastern respiratory syndrome coronavirus and Zika virus” with spending totaling near 500,000 USD between 2016 and 2021. The International Vaccine Research Institute completed their project, “Discovery of MERS-CoV vaccine Candidate and Evaluation of Vaccine efficacy using in vitro system”, having spent 318,182 USD between 2015 and 2019 (see Table 9).

Thirdly, the platform for detection and point-of-care diagnostics in Korea (Sub-Cluster 1-3) is composed of 45 projects totaling 23,872,321 USD. Chonbuk National University started conducting their project “The development of prompt and customized genetic engineering technology-based approaches for the control of disastrous infectious diseases” in 2017, which is expected to finish in 2026 with total expenditures of 477,328 USD. Meanwhile, The Korea Research Institute of Bioscience and Biotechnology’s project “Next-generation virus detection and control technology development” has a budget of 87,273 USD between 2019 and 2023. In the for-profit sector, Seoul’s Asan Hospital has dedicated 153,182 USD towards their project, “Advancement of specimen processing and evaluation of ultrasensitive diagnostic platform technologies for emerging viruses” between 2016 and 2021 (see Table 10).

#### 3.2.2. Research on the Virus Protein Structure, Activity/Function-Based Antivirus Therapeutics (Treatment) Design, and Activity/Resistance Modulation in Korea (Cluster 2)

Overall, research on the virus protein structure, activity/function-based antivirus therapeutics (treatment) design, and activity/resistance modulation in Korea (Cluster 2) consisted of 32 projects, totaling 14,451,688 USD.

Firstly, structure–activity relationship modeling-based virus prediction and activity modulation in Korea (Sub-Cluster 2-1) is composed of seven projects, worth 2,775,533 USD. The Korea Research Institute of Bioscience and Biotechnology has committed to funding a project entitled “Development of viral recombination prediction and validation technique using bioinformatics” with projected expenditures of 272,727 USD, which started in 2014 and is projected to finish in 2022. Yonsei University’s “Basic research on new drug candidate discovery based on chemo-informatics” project spent 890,909 USD between 2006 and 2009. Meanwhile, Kyungdon University’s project, “Big-data analysis on viral infection using epigenetic information”, spent 144,475 USD between 2016 and 2019 (see Table 11).

Secondly, studies on the design of antiviral agents based on the structure and function of viral and human receptor proteins in Korea (Sub-Cluster 2-2) were composed of 25 projects worth 11,676,155 USD. Institut Pasteur Korea has pledged to spend 3 million USD on the project entitled “Preparedness of emerging viruses” between 2017 and 2022. Ilyang Pharmaceutical Co., Ltd. and Hallym University also have concentrated on a similar project, “Deriving new candidates for the development of therapeutics in the Middle East Respiratory Syndrome”, with budgeted expenditures of 763,636 and “Development therapeutic target and candidate for immunotherapy against middle east respiratory syndrome coronavirus (MERS-CoV)” with a budget of 418,182 USD between 2016 and 2021, respectively (see Table 12).

#### 3.2.3. Infectious Disease Epidemiological Investigation and Animal and Environmental Ecology in Korea (Cluster 3)

Infectious disease epidemiological investigation and animal and environmental ecology in Korea (Cluster 3) contained 12 projects worth 2,589,259 USD. Seoul City University is executing a 227,273 USD project entitled “Studies on the Development of MERS Diffusion Route Detection and Prevention Technology”, which started in 2019 and is expected to be completed by 2024. Meanwhile, the National Medical Center finished “A Four year follow-up clinical and immunological study of MERS patients” project worth 181,816 USD (see Table 13).

### 3.3. Comparison between the Developed Nations and Korea

Due to the differences in the absolute amount of R&D funding per nation, ratio analysis was undertaken to compare the relative magnitudes of these coronavirus-related R&D areas among nations, which allowed for the identification of these nations’ strengths and weaknesses [32].

**Figure 3:**
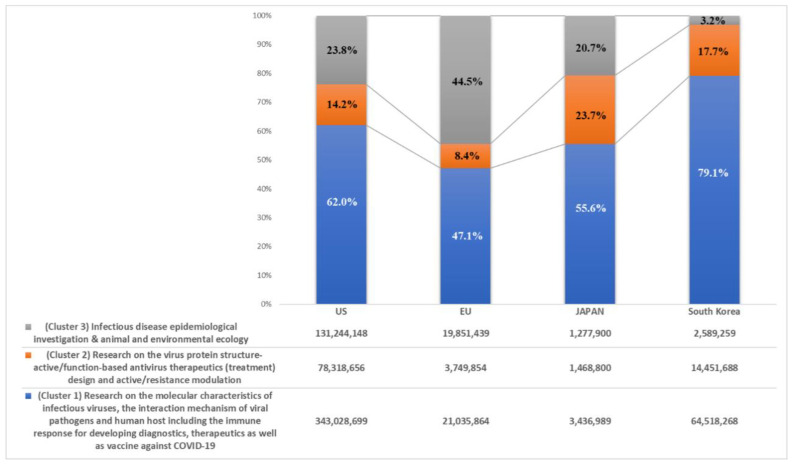
Comparative ratio analysis for three coronavirus-related R&D areas among the US, the EU, Japan, and Korea.

The US has a wide range of research fields for infectious disease preparedness ranging from basic research (i.e., the identification and mechanism of the viral pathogen) (62.0%) to animal–environmental ecology and the epidemiologic investigation and quarantine of infectious diseases (23.8%) and has invested from a mid- to long-term perspective (see Figure 3). Although the funding scale for coronavirus-related R&D areas in the EU and Japan is smaller than that of the United States, the directions for the R&D areas are considerably analogous to those in the US. These trends in coronavirus-related R&D funding show that the directions for the R&D investment are in accordance with the “One Health” concept of the WHO, which is a global strategy for all aspects of healthcare for the environment–animal–human paradigm [33].

**Figure 4:**
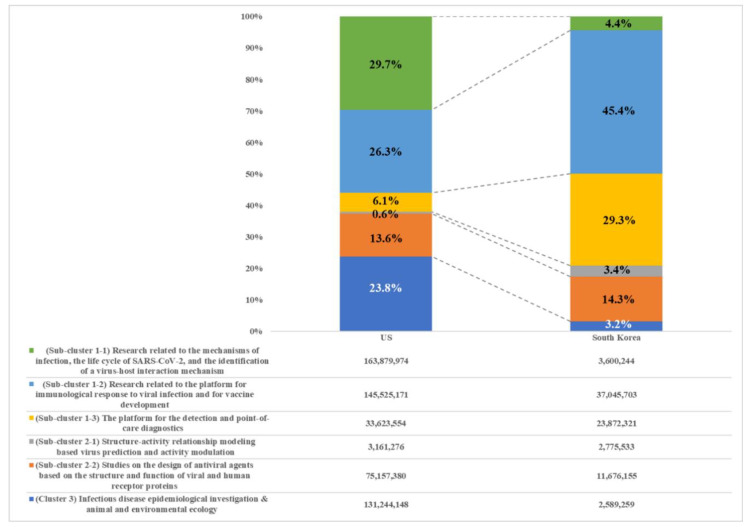
Comparative ratio analysis for six coronavirus-related R&D areas for the US and Korea.

The Korean government has invested in coronavirus-related basic research and core technologies (Sub-Cluster 1-1: 4.4%, Sub-Cluster 1-2: 45.4%, and Sub-Cluster 1-3: 29.3%), including the identification of infectious disease pathogens, the structure and replication mechanism of viruses, infection mechanisms in the host, and immune responses, which are dominated by the US and EU’s technological edge. However, some coronavirus-related R&D areas were heavily supported by the Korean government compared to the US. These R&D areas covered research related to the platform for the immunological response to viral infections and for vaccine development (Sub-Cluster 1-2: Korea: 45.4% vs. the US: 26.3%), the platform for detection and point-of-care diagnostics (Sub-Cluster 1-3: Korea: 29.3% vs. the US: 6.1%), and the studies on the design of antiviral agents based on the structure and function of viral and human receptor proteins (Sub-Cluster 2-2: Korea: 14.3% vs. the US: 13.6%), which are not only highly applied in clinical settings in practice but also have high possibilities for technological commercialization from a short- or medium-term perspective (see Figure 4). The characteristics of Korea’s R&D funding may be due to the combined needs of the profit sector, which has emphasized short- or medium-term returns on investment, and the bio-industry promotion policy of the Korean government to strengthen the private sector’s technological advantage. Moreover, it is reasonable to deduce that the government-driven R&D on viruses after the spread of MERS-CoV in 2015 also had a profound impact on expenditures.

## 4. Discussion 

This study aimed to elucidate the trends of coronavirus-related R&D—because COVID-19 has been globally endangering human health and well-being since December 2019 and, at the moment, the spread of COVID-19 has barely been controlled in Korea—thereby deriving directions for government-driven R&D to prepare for the post-corona pandemic era. Three coronavirus-related R&D areas were clustered through co-occurrence matrix analysis, and then, on the subjects of two of them, five sub-clusters were additionally extrapolated based on experts’ reviews. According to the results, the US, the EU, and Japan have invested in coronavirus-related research areas through 160 projects worth 1,099,242,389 USD, nine projects worth 44,637,157 USD, and 46 projects worth 6,183,689 USD, respectively. Meanwhile, Korea has funded 170 projects worth about 81,559,215 USD. Due to the centralized database of Korea, its coronavirus-related R&D activities may be overestimated. Despite this limitation, we found that developed nations focused their propensity on investing in entire research areas. On the other hand, due to the outbreak of MERS-CoV in 2015, Korea is likely to continue pursuing technological commercialization to strengthen its bio-industry competitiveness in some specific areas rather than basic research areas from a short-and medium-term viewpoint.

## 5. Conclusions

This result has three profound implications for Korea. Firstly, the coronavirus crisis has affected the healthcare systems of Korea locally and has driven many countries around the world to their breaking points, thereby making it clear that the international collaboration of the entire R&D network must remain a priority. Thus, the Korean government must confront the deficiency of their capabilities in core technology areas rather than the “K-quarantine” model, which was emphasized as an asset to support economic cooperation, to pioneer new markets, and to bolster the nation’s reputation in the international community [34]. Secondly, it is useful for Korea to establish long-term strategies such as international cooperation programs in human resource exchange and bilateral/multilateral R&D activities among the leading organizations. In particular, it is necessary to develop the infectious disease-related basic research, diagnostics, vaccines, and therapeutics where Korea’s technology is insufficient. Finally, basic information on the amount of R&D funding and research organizations in leading nations may allow stakeholders in these nations to consider the future directions for establishing R&D investments and policies in the post-corona crisis era. National funding data-based analysis could only provide financial information, which was impossible to deduce when a scientific publication- or patent-based analysis was undertaken.

As the coronavirus crisis has had devastating impacts on all social and economic sectors around the world, there is a strong need for the adequate global detection and an adequate response to the identification of new variants, including new epidemic variants or new variants with pandemic potential, by enhancing globally collaborative efforts in scientific societies. It is necessary to investigate the status quo of the R&D of global infectious diseases in order to improve global cooperation strategies, with more effective organizational support through funding the R&D of technologies that deal with infectious diseases. Further study is needed on the analysis of R&D funding in virology, recognized as a basic research area for battling against infectious diseases more effectively, and its associated disciplines to strengthen the capacity of global infectious disease surveillance. It may provide useful insights for strategic approaches to an international collaboration network, thereby enhancing the assessment of international cooperative research projects and improving the capacity for a global response to infectious disease threats.

As aforementioned, the US, the EU, and Japan’s data sources did not include information on the entire funding data of these nations. However, Korea’s NTIS contains a centralized database for the entire data of national funding, thereby creating a “home advantage” bias. Moreover, the limitation of this study was that it was focused narrowly on coronavirus-related national funded projects. Thus, the data that were retrieved could not cover the study of other serious infectious diseases such as Zika, Ebola, and Nipah Virus.

## Figures and Tables

**Table 1:** The source of global R&D project data.

Geographic Region	Data Source	Search Terms	Number of Raw Data	Number of Utilized Data
USA— STAR-METRICS	Global R&D database	(corona * AND virus *) OR coronavirus * OR mers-cov * OR sars-cov * OR covid * OR “severe acute respiratory syndrome” OR “middle east respiratory syndrome”)	554	228
EU—CORDIS			35	10
Japan—KAKEN			10	35
Korea	NTIS	(corona * | sars * | mers *)	638	170
		Total (Period: 2012–2018)	1237	443

Search terms marked with an asterisk (*) are root or stem words, indicating that all possible suffixes are covered under the query.

**Table 2:** Research related to the mechanisms of infection, the life cycle of SARS-CoV-2, and the identification of a virus–host interaction mechanism (Sub-Cluster 1-1).

NO	Organization	Title	Estimated Average Cost/Fiscal Year (USD)	Estimated Total Cost (USD)	Start Date	End Date	Geographic Region
1	University of Maryland Baltimore	Host, pathogen, and the microbiome: determinants of infectious disease outcomes	3,926,048	19,630,240	15 April 2014	31 March 2019	US
2	The University of Pittsburgh	Targeting host responses to prevent virus induced ARDS in the nonhuman primate model	1,809,375	1,809,375	15 September 2018	14 September 2021	US
3	The University of Iowa	Role of eicosanoids in pathogenic human CoV infections	545,235	2,726,175	23 September 2016	31 August 2021	US
4	Erasmus Universitair Medisch Centrum Rotterdam	European management platform for emerging and reemerging infectious disease entities	3,736,307	18,681,536	1 May 2009	30 April 2014	EU
5	Yamaguchi University	Elucidation of the mutation mechanism and virulence acquisition machine of coronavirus	171,600	514,800	1 April 2015	31 March 2018	JP

**Table 3:** Research related to the platform for an immunological response to viral infection and vaccine development (Sub-Cluster 1-2).

NO	Organization	Title	Estimated Average Cost/Fiscal Year (USD)	Estimated Total Cost (USD)	Start Date	End Date	Geographic Region
1	University of North Carolina Chapel Hill	Systems immunogenetics of biodefense and emerging pathogens in the collaborative cross	2,437,629	24,376,290	5 August 2012	31 August 2022	US
2	New York Blood Center	Structure-based design of coronavirus subunit vaccines	911,083	4,555,415	21 May 2018	30 April 2023	US
3	Planet Biotechnology Inc	A MERS-CoV receptor decoy	600,081	2,400,324	1 June 2014	31 January 2018	US
4	University of Pennsylvania	Murine coronavirus neurovirulence role of type i interferon response	341,731	2,392,117	1 September 2012	31 May 2019	US
5	National Institute of Infectious Diseases	The development of a new vaccine against human coronavirus that causes severe pneumonia	48,100	144,300	1 April 2016	31 March 2019	JP

**Table 4:** The platform for detection and point-of-care diagnostics (Sub-Cluster 1-3).

NO	Organization	Title	Estimated Average Cost/Fiscal Year (USD)	Estimated Total Cost (USD)	Start Date	End Date	Geographic Region
1	Columbia University Health Sciences	Diagnostic and prognostic biomarkers for severe viral lung disease	1,131,261	5,656,305	7 March 2014	28 February 2019	US
2	Boston College	New genetic tools for comparative analysis of emerging viruses and virus host molecular interactions in reservoir hosts versus spillover hosts	313,000	626,000	1 April 2018	30 September 2019	US
3	Crosslife Technologies, Inc.	SBIR phase 1: rapid instrument-free nucleic acid test for pathogens and biothreats	224,929	224,929	1 June 2017	31 May 2018	US
4	Erasmus Universitair Medisch Centrum Rotterdam	Early detection of emerging viruses by next generation in situ hybridization	102,946	205,891	1 July 2014	30 June 2016	EU
5	Nihon University	Establishment of gene diagnosis of feline infectious peritonitis by the analysis of the viral genome	44,200	132,600	1 April 2015	31 March 2018	JP

**Table 5:** Structure–activity relationship modeling-based virus prediction and activity modulation (Sub-Cluster 2-1).

NO	Organization	Title	Estimated Average Cost/Fiscal Year (USD)	Estimated Total Cost (USD)	Start Date	End Date	Geographic Region
1	Blood Systems Research Institute	Modeling viral entry and its inhibition using SARS-CoV	406,843	2,034,215	15 December 2007	30 November 2012	US
2	Harvard University	Structure and mechanism of programmed ribosomal frameshifting in SARS coronavirus	375,687	1,127,061	7 February 2013	31 January 2016	US
3	Katholieke Universiteit Leuven	Virus discovery and epidemic tracing from high throughput metagenomic sequencing	1,168,428	3,505,285	1 June 2015	31 May 2018	EU
4	Osaka University	Elucidation of the establishment and the virulence factors of reverse genetic manipulation system of MERS coronavirus	48,100	144,300	1 April 2016	31 March 2019	JP
5	Yamaguchi University	Analysis of coronavirus evolution mechanism for appearance prediction emerging coronavirus	21,700	43,400	24 April 2015	31 March 2017	JP

**Table 6:** Studies on the design of antiviral agents based on the structure and function of viral and human receptor proteins (Sub-Cluster 2-2).

NO	Organization	Title	Estimated Average Cost/Fiscal Year (USD)	Estimated Total Cost (USD)	Start Date	End Date	Geographic Region
1	University of North Carolina Chapel Hill	Broad-spectrum antiviral GS-5734 to treat MERS-CoV and related emerging CoV	1,310,955	6,554,775	9 August 2017	31 July 2022	US
2	University of Alabama at Birmingham	Inhibitors of coronavirus fidelity and cap methylation as broadly applicable	996,397	4,981,985	1 March 2014	28 February 2019	US
3	Kansas State University	Small molecule protease inhibitors against MERS-CoV	775,916	3,879,580	15 May 2018	30 April 2023	US
4	The Johns Hopkins University	Applying human factors and mathematical modeling approaches to prevent transmission of high consequence pathogens	1,222,700	3,668,100	30 September 2015	29 September 2018	US
5	Nippon Veterinary and Life Science University	The development of specific anti coronavirus drugs using the novel glycosidase inhibitors	48,100	144,300	1 April 2016	31 March 2019	JP

**Table 7:** Infectious Disease Epidemiological Investigation and Animal and Environmental Ecology (Cluster 3).

NO	Organization	Title	Estimated Average Cost/Fiscal Year (USD)	Estimated Total Cost (USD)	Start Date	End Date	Geographic Region
1	George Washington University	Ecology of MERS-CoV in camels, humans, and wildlife in Ethiopia	2,487,071	9,948,284	1 September 2018	31 August 2022	US
2	Montana State University—Bozeman	Dynamics of zoonotic systems: human-bat- pathogen interactions	1,650,000	6,600,000	1 September 2017	31 August 2021	US
3	Fred Hutchinson Cancer Research Center	Realtime tracking of virus evolution for vaccine strain selection and epidemiological investigation	409,400	2,047,000	23 August 2016	31 May 2021	US
4	Erasmus Universitair Medisch Centrum Rotterdam	Anticipating the global onset of novel epidemics	3,670,585	18,352,925	1 November 2011	31 October 2016	EU
5	National Institute of Infectious Diseases	The survey of middle east respiratory syndrome coronavirus of the dromedary in Ethiopia	137,800	413,400	1 April 2017	31 March 2020	JP

**Table 8:** Research related to the mechanisms of infection, the life cycle of SARS-CoV-2, and the identification of a virus–host interaction mechanism in Korea (Sub-Cluster 1-1).

NO	Organization	Title	Estimated Average Cost/Fiscal Year (USD)	Estimated Total Cost (USD)	Start Date	End Date
1	Kookmin University	Reaction mechanism study on SARS Coronavirus helicase and its applications to develop the inhibitors	45,736	137,209	1 November 2013	31 October 2016
2	Chonbuk National University	A genetic characteristic study for zoonotic potential of several pathogens from bats in Korea	15,152	45,455	1 June 2018	31 May 2021
3	Kookmin University	Mechanistic study on SARS Coronavirus helicase in the process of viral genome replication	18,272	54,816	1 September 2010	31 August 2013
4	Chungnam National University	Identification of mechanism of coronavirus cross-species transmission and pathogenesis mediated by host proteases	45,455	136,364	1 March 2020	28 February 2023
5	Center for Disease Control	Immunogenocity of SARS-CoV receptor binding protein expressed on coxsackieviral vector	30,455	60,909	1 January 2010	31 December 2011

**Table 9:** Research related to the platform for the immunological response to viral infection and for vaccine development in Korea (Sub-Cluster 1-2).

NO	Organization	Title	Estimated Average Cost/Fiscal Year (USD)	Estimated Total Cost (USD)	Start Date	End Date
1	Seoul National University	The development of therapeutic antibodies against Middle East respiratory syndrome coronavirus and Zika virus	99,273	496,364	1 July 2016	31 March 2021
2	International Vaccine Research Institute	Discovery of MERS-CoV vaccine Candidate and Evaluation of Vaccine efficacy using in vitro system	79,545	318,182	3 December 2015	30 November 2019
3	Korea University	Development of next-generation universal vaccine to control viral infectious diseases	181,818	181,818	1 December 2015	30 November 2016
4	Center for Disease Control	Expression and immunogenicity study of Coxsackievirus vector of SARS-CoV receptor binding protein	28,788	86,364	1 January 2008	31 December 2010
5	Chonbuk National University	Development of standard protocol for measuring the amount of vaccine candidate and the titer of antibody for MERS-CoV(2)	22,727	45,455	16 February 2016	30 November 2017

**Table 10:** The platform for detection and point-of-care diagnostics in Korea (Sub-Cluster 1-3).

NO	Organization	Title	Estimated Average Cost/Fiscal Year (USD)	Estimated Total Cost (USD)	Start Date	End Date
1	Chonbuk National University	The development of prompt and customized genetic engineering technology-based approaches for the control of disastrous infectious diseases	53,036	477,328	1 June 2017	28 February 2026
2	Konkuk University	Development of platform technology for highly sensitive detection of emerging viruses using gene amplification and fluorescence monitoring	48,182	240,909	1 July 2016	31 March 2021
3	Seoul Asan Hospital	Advancement of Specimen Processing and Evaluation of Ultrasensitive Diagnostic Platform Technologies for Emerging Viruses	30,636	153,182	1 July 2016	31 March 2021
4	Korea Research Institute of Bioscience and Biotechnology	Next-generation virus detection and control technology development	17,455	87,273	1 January 2019	31 December 2023
5	Center for Disease Control	Development of diagnostic resources for the rapid response of emerging respiratory viruses	181,818	545,455	1 January 2016	31 December 2018

**Table 11:** Structure–activity relationship modeling-based virus prediction and activity modulation in Korea (Sub-Cluster 2-1).

NO	Organization	Title	Estimated Average Cost/Fiscal Year (USD)	Estimated Total Cost (USD)	Start Date	End Date
1	Korea Research Institute of Bioscience and Biotechnology	Development of viral recombination prediction and validation technique using bioinformatics	30,303	272,727	1 January 2014	31 August 2022
2	Yonsei University	Basic research on new drug candidate discovery based on chemo-informatics	296,970	890,909	1 September 2006	31 August 2009
3	Seoul National University	Simulation study for the prediction of zoonotic infection risk using genetic variation markers of viruses	26,515	79,545	1 June 2017	31 May 2020
4	Kyungdong University	Big-data analysis on viral infection using epigenetic information	48,158	144,475	1 June 2016	31 May 2019
5	Center for Disease Control	Variation predict and viral attenuation of MERS-CoV	254,545	763,636	1 January 2016	31 December 2018

**Table 12:** Studies on the design of antiviral agents based on the structure and function of viral and human receptor proteins in Korea (Sub-Cluster 2-2).

NO	Organization	Title	Estimated Average Cost/Fiscal Year (USD)	Estimated Total Cost (USD)	Start Date	End Date
1	Institut Pasteur Korea	Preparedness of Emerging Viruses	500,035	3,000,210	30 June 2017	31 December 2022
2	Ilyang Pharmaceutical Co., Ltd.	Deriving new candidates for the development of therapeutics in the Middle East Respiratory Syndrome	152,727	763,636	1 July 2016	31 March 2021
3	Hallym University	Development of therapeutic target and candidate for immunotherapy against middle east respiratory syndrome coronavirus (MERS-CoV)	83,636	418,182	1 July 2016	31 March 2021
4	Korea Advanced Institute of Science and Technology	Development of novel therapeutics for infectious diseases through activation of innate immune system	178,182	356,364	1 November 2015	31 October 2017
5	Chonbuk National University	Development of recombinant subunit vaccine candidates for the protection against MERS-CoV infection	68,182	272,727	3 December 2015	30 November 2019

**Table 13:** Infectious disease epidemiological investigation and animal and environmental ecology in Korea (Cluster 3).

NO	Organization	Title	Estimated Average Cost/Fiscal Year (USD)	Estimated Total Cost (USD)	Start Date	End Date
1	Seoul City University	Studies on the Development of MERS Diffusion Route Detection and Prevention Technology: focus on public transportation users	45,455	227,273	29 November 2019	28 November 2024
2	Inha University	Mathematical Control Strategies for Effective Preventive Measures Against Epidemics: Macroscopic and Microscopic Viewpoints	26,515	79,545	1 June 2017	31 May 2020
3	Konkuk University	Mathematical models of contact management and spread of infectious diseases	90,909	181,818	7 November 2018	31 December 2019
4	The National Medical Center	A Four year follow-up clinical and immunological study of MERS patients	181,816	181,816	1 February 2019	31 December 2019
5	Woori Airtech Korea Co., Ltd.	Developed mobile sound pressure booths to prevent secondary infections and medical staff when treating various infectious diseases such as MERS	58,237	58,237	1 June 2016	28 February 2017

## Supplementary Materials

The following are available online at https://www.mdpi.com/2227-9032/8/3/204/s1. Table S1: Research related to mechanisms of infection, the life cycle of SARS-CoV-2, and identification of virus-host interaction mechanism (Sub-cluster 1-1), Table S2: Researches related to platform for immunological response to viral infection and for vaccine development (Sub-cluster 1-2), Table S3: Platform for detection and point-of-care diagnostics (Sub-cluster 1-3), Table S4: Structure-activity relationship modeling based virus prediction and activity modulation (Sub-cluster 2-1), Table S5: Studies on design of antiviral agents based on the structure and function of viral and human receptor proteins in Korea (Sub-cluster 2-2), Table S6: Infectious Disease Epidemiological Investigation & Animal and Environmental Ecology (Cluster 3), Table S7: Researches related to mechanisms of infection, the life cycle of SARS-CoV-2, and identification of virus-host interaction mechanism in Korea (Sub-cluster 1-1), Table S8: Researches related to platform for immunological response to viral infection and for vaccine development in Korea (Sub-cluster 1-2), Table S9: Platform for detection and point-of-care diagnostics in Korea (Sub-cluster 1-3), Table S10: Structure-activity relationship modeling based virus prediction and activity modulation in Korea (Sub-cluster 2-1), Table S11: Studies on design of antiviral agents based on the structure and function of viral and human receptor proteins in Korea (Sub-cluster 2-2), Table S12: Infectious Disease Epidemiological Investigation & Animal and Environmental Ecology in Korea (Cluster 3).
