# Supplementary material for: A Strategy for International Cooperation in the COVID-19 Pandemic Era: Focusing on National Scientific Funding Data
Source: Healthcare (Basel). 2020 Jul 9;8(3):204. doi: 10.3390/healthcare8030204 (PMC7551450; doi:10.3390/healthcare8030204)
Supplement: Supplementary file 1 [file healthcare-08-00204-s001.pdf]

healthcare MDPI

Supplementary Materials: Coronavirus-related R&D areas of the United States, the European Union, Japan, and South Korea.

Table S1. Research related to mechanisms of infection, the life cycle of SARS-CoV-2, and identification of virus-host interaction mechanism (Sub-cluster 1-1).

NO	Organization	Title	Estimated Average Cost/Fiscal Year (USD)	Estimated Total Cost (USD)	Start date	End date	Geographic Region
1	University of Maryland Baltimore	Host-pathogen and the microbiome: determinants of infectious disease outcome	3,926,048	19,630,240	15 April 2014	31 March 2019	US
2	Children's Hospital Boston	Structure and assembly of viruses	379,571	14,423,698	1 June 1975	30 April 2013	US
3	University of Tennessee Knoxville	Coronavirus RNA replication	349,845	12,944,265	1 July 1977	31 May 2014	US
4	University of North Carolina Chapel Hill	Characterization of novel genes encoded TY RNA and DNA viruses	2,117,960	12,707,760	21 June 2013	31 May 2019	US
5	University of Pennsylvania	Molecular biology of coronavirus induced demyelination	299,302	9,278,362	1 July 1985	30 November 2015	US
6	Vanderbilt University	Polymerase proteins in coronavirus replication	284,420	6,826,080	30 September 1991	30 April 2015	US
7	Wadsworth Center	Genetic analysis of molecular interactions in coronavirus replication	517,899	5,696,889	1 June 2005	31 March 2016	US
8	Vanderbilt University	The cell biology of coronavirus infection	343,312	4,463,056	15 December 2002	30 November 2015	US
9	University of North Carolina Chapel Hill	Mechanisms of MERS-CoV entry cross species transmission and pathogenesis	738,577	3,692,885	20 April 2015	31 March 2020	US

10	University of North Carolina Chapel Hill	Molecular biology of viral diseases	179,474	3,589,480	30 September 1993	31 August 2013	US
11	Northwestern University at Chicago	Barrier integrity microbiome and HIV target cell interactions in the human male genital tract pre and post circumcision	638,625	3,193,125	1 September 2017	31 July 2022	US
12	Loyola University Chicago	Mechanisms of viral proteases in coronavirus replication and pathogenesis	627,215	3,136,075	1 July 2010	30 June 2015	US
13	Ecohealth Alliance Inc	Understanding the risk of bat coronavirus emergence	617,347	3,086,735	1 June 2014	31 May 2019	US
14	Oregon Health and Science University	Systems biology approaches to viral pathogenesis and immunity	960,510	2,881,530	1 March 2011	28 February 2014	US
15	Lovelace Biomedical & Environmental Research Institute Inc	SARS coronavirus virulence factors and lung pathogenesis	471,906	2,831,436	3 April 2006	31 March 2012	US
16	University of Maryland Baltimore	Targeting TLR signaling pathways to blunt pathogen mediated acute lung injury	547,143	2,735,715	10 February 2017	31 January 2022	US
17	University of Pennsylvania	Neurovirulence of murine coronavirus roles of viral genes	389,837	2,728,859	3 April 2006	31 March 2013	US
18	University of Iowa	Role of eicosanoids in pathogenic human CoV infections	545,235	2,726,175	23 September 2016	31 August 2021	US
19	University of California Irvine	Molecular biology of eukaryotic viruses	100,277	2,607,202	30 September 1988	30 November 2013	US
20	University of Pennsylvania	MERS coronavirus antagonism of double stranded RNA induced host response by accessory proteins	497,163	2,485,815	24 May 2018	30 April 2023	US
21	University of North Carolina Chapel Hill	SARS-CoV pathogenic mechanisms in senescent mice	488,268	2,441,340	1 April 2008	31 March	US

						2013	
22	Oregon Health and Science University	Systems pathogenomics of severe acute respiratory virus infection	758,603	2,275,809	1 March 2011	28 February 2014	US
23	University of Utah	Strategies to prevent infection and reduce interindividual transmission spirit	733,333	2,200,000	30 September 2015	29 September 2018	US
24	University of Iowa	Biology of ace2 activity in the airway epithelium	302,719	2,119,033	19 June 2011	15 August 2017	US
25	Cornell University Ithaca	Structural and functional analysis of the coronavirus spike protein fusion peptide	506,453	2,025,812	9 August 2018	31 July 2022	US
26	University of North Carolina Chapel Hill	Role of uncharacterized genes in high pathogenic human coronavirus infection	396,549	1,982,745	1 June 2012	31 October 2016	US
27	University of Maryland Baltimore	Role of the epithelial growth factor receptor in SARS coronavirus pathogenesis	376,852	1,884,260	1 July 2011	30 June 2016	US
28	U of L Imperial Col of Sci/Technology/Med	Models for synthesizing molecular clinical and epidemiological data and translate	370,861	1,854,305	15 September 2014	31 May 2019	US
29	The University of Pittsburgh	Targeting host responses to prevent virus induced ARDS in the nonhuman primate model	1,809,375	1,809,375	15 September 2018	14 September 2021	US
30	Indiana University Bloomington	Novel RNA structures in coronavirus replication	356,180	1,780,900	1 January 2008	31 December 2012	US
31	University of Texas Medical Br Galveston	Analysis of SARSs coronavirus host cell interactions	291,833	1,750,998	5 August 2007	31 July 2013	US
32	University of Texas Medical Br Galveston	New paradigm for host and viral gene regulation by MERS coronavirus nsp1	346,482	1,732,410	1 May 2015	30 April 2020	US

33	University of Iowa	Entry and pathogenesis of two human coronaviruses	338,636	1,693,180	19 July 2011	30 June 2016	US
34	Vanderbilt University	Determinants of coronavirus fidelity in replication and pathogenesis	481,881	1,445,643	1 August 2013	30 April 2016	US
35	University of Texas Medical Br Galveston	Analysis of coronavirus host cell interactions	329,960	1,319,840	15 March 2012	29 February 2016	US
36	University of Washington	Structural studies of coronavirus fusion proteins	259,841	1,299,205	1 August 2016	31 July 2021	US
37	Harvard University	Identification and characterization of cellular receptors for enveloped viruses	418,975	1,256,925	1 March 2011	28 February 2014	US
38	University of Northern Colorado	Experimental tacrine virus infection of Jamaican fruit bats	402,668	1,208,004	19 July 2010	30 June 2013	US
39	University of Pennsylvania	Pathogenesis of murine coronavirus induced hepatitis	400,000	1,200,000	30 September 2011	29 September 2014	US
40	Loyola University Chicago	Entry and pathogenesis of coronaviruses	376,191	1,128,573	1 September 2011	31 August 2014	US
41	University of Missouri-Columbia	Restriction of viral membrane fusion and entry by infirm proteins	392,973	785,946	4 August 2014	31 July 2016	US
42	University of Pennsylvania	Mechanism of murine coronavirus induced hepatitis	236,250	708,750	1 June 2009	31 May 2012	US
43	Texas A & M University Health Science Ctr	A genetic analysis of pneumotropism and pneumovirulence in an mhv1 model of SARS	224,284	672,852	22 May 2009	30 April 2012	US
44	Children's Hospital Boston	Viral and host determinants of new world alpha viral entry	133,515	667,575	1 June 2011	31 May 2016	US
45	Drexel University	Role of microRNAs in coronavirus induced lethal encephalitis	212,407	637,221	1 June 2011	31 May 2014	US
46	Loyola University Chicago	Ubiquitin and cellular factors in coronavirus assembly	186,875	560,625	5 June 2009	31 May 2012	US

47	University of Pennsylvania	Control of coronavirus pathogenesis by antagonism of RNAse l	227,125	454,250	1 July 2015	30 June 2017	US
48	Bucknell University	Transcriptomics of immunity and disease in African fruit bats important zoonotic reservoirs	220,229	440,458	1 January 2017	31 December 2018	US
49	University of Texas Medical Br Galveston	Establishment of a transgenic mouse model for middle east respiratory syndrome co	213,000	426,000	1 June 2014	31 May 2016	US
50	Texas A & M University Health Science Ctr	Conserved RNA secondary structures in three beta coronaviruses MHV CoV and MERS-CoV	211,875	423,750	15 March 2016	28 February 2018	US
51	University of Maryland College Park Campus	New paradigms for ribosome recoding in strand viruses	204,978	409,956	1 February 2015	31 January 2017	US
52	University of Wisconsin Madison	Flow enhanced characterization of drug resistant viruses	98,964	395,856	15 September 2008	30 June 2012	US
53	University of North Carolina Chapel Hill	Systems based analysis of host factors that contribute to aging pathogenesis	150,663	301,326	15 July 2015	31 May 2017	US
54	Scripps Research Institute	Structural studies of the coronavirus life cycle	103,478	206,956	14 July 2017	30 June 2019	US
55	Drexel University	Role of toll like receptors 2 and 3 in coronavirus induced encephalitis	76,168	152,336	1 April 2010	31 March 2012	US
56	University of North Carolina Chapel Hill	Evaluation of SARS-CoV 2o methyltransferase mutants	44,030	132,090	1 January 2013	31 December 2015	US
57	Vanderbilt University	Roles of replication fidelity in viral RNA synthesis population diversity and overall fitness of coronaviruses	28,952	115,808	13 January 2017	12 January 2021	US
58	University of Pennsylvania	Investigating MERS-CoV ns4b as a modulator of the host antiviral response in the nucleus	37,817	113,451	1 September 2017	31 August 2020	US
59	University of Kentucky	Fusion protein TMTM interactions modulation of prefusion protein stability	34,703	69,406	15 May 2015	14 May 2017	US
60	University of Iowa	Deciphering the role of the coronavirus macro domain in SARS-CoV infection	53,282	53,282	25 December	24 December	US

					2014	2015	
61	University of Pennsylvania	Coronavirus antagonism of the oasRNAse l pathway	46,470	46,470	1 January 2015	31 October 2015	US
62	Cornell University Ithaca	Mouse hepatitis coronavirus receptor binding domain	10,975	10,975	1 April 2011	31 March 2012	US
63	Keystone Symposia	Positive strand RNA viruses	8,000	8,000	8 March 2013	28 February 2014	US
64	Loyola University Chicago	Nido2017 international norovirus symposium	5,000	5,000	1 June 2017	31 May 2018	US
65	Keystone Symposia	Pathogenesis of respiratory viruses	5,000	5,000	1 January 2014	31 December 2014	US
66	Cornell University Ithaca	Internal fusion peptide of the severe acute respiratory syndrome coronavirus	2,896	2,896	1 September 2011	31 August 2012	US
67	Research Institute, International Medical Center of Japan	Mode of severe respiratory failure by SARS-CoV	184,600	553,800	1 January 2010	31 December 2012	Japan
68	Yamaguchi University	Elucidation of the mechanism mutation mechanism and virulence acquisition machine of coronavirus	171,600	514,800	1 April 2015	31 March 2018	Japan
69	Fujita Health University	The clustering mechanism and the driving force of the macro membrane domains	54,600	163,800	1 April 2012	31 March 2015	Japan
70	National Institute of Agrobiologically Sciences	A study on the virus superfamily 1 helicase structure and function of the towards drug discovery and development	52,000	156,000	1 April 2013	31 March 2016	Japan
71	National Institute of Infectious Diseases	Construction of the foundation technologies for the MERS coronavirus natural host animal identification	49,400	148,200	1 April 2014	31 March 2017	Japan
72	Soka University	Pathogenesis of spongiform encephalopathy induced by viral infection	46,800	140,400	1 January 2010	31 December 2012	Japan

73	Nippon Veterinary and Life Science University	Assembly mechanisms of coronaviruses	41,600	124,800	1 April 2013	31 March 2016	Japan
74	Osaka University	Understanding of viral replication through the interaction with coronavirus nsp1 and host proteins	44,200	88,400	1 April 2012	31 March 2014	Japan
75	University of Yamanashi	The analysis of the mechanism of SARS coronavirus nsp1mediated mRNA decay	41,600	83,200	1 April 2013	31 March 2015	Japan
76	Juntendo University	Role of cd26 molecule in the infection of MERS and development of new therapy	36,400	72,800	1 April 2014	31 March 2016	Japan
77	National Agriculture and Food Research Organization	Elucidation of verification and virus dynamics of persistent infection in cattle body of cattle coronavirus	54,600	54,600	1 April 2012	31 March 2013	Japan
78	Erasmus Universitair Medisch Centrum Rotterdam	European management platform for emerging and reemerging infectious disease entities	3,736,307	18,681,536	1 May 2009	30 April 2014	EU
79	Universitat De Valencia	Variability in the mutation rate of RNA viruses	335,093	1,675,465	1 January 2012	31 December 2016	EU
80	Universidad Politecnica De Madrid	Analysis of speciation mechanisms in RNA viruses	84,564	253,691	1 July 2009	30 June 2012	EU
81	Eidgenoessisches Departement Des Innern	Unravelling species barriers of coronaviruses	109,641	219,281	1 May 2017	30 April 2019	EU

Table S2. Researches related to platform for immunological response to viral infection and for vaccine development (Sub-cluster 1-2).

NO	Organization	Title	Estimated Average Cost/Fiscal Year (USD)	Estimated Total Cost (USD)	Start date	End date	Geographic Region
1	University of North Carolina Chapel Hill	Systems immunogenetics of biodefense and emerging pathogens in the collaborative cross	2,437,629	24,376,290	5 August 2012	31 August 2022	US
2	University of Iowa	Pig SARS-COV host cell interactions and vaccine development	1,598,570	19,182,840	1 August 2004	30 June 2016	US
3	University of North Carolina Chapel Hill	Systems immunogenetics of biodefense pathogens in the collaborative cross	4,354,308	17,417,232	5 August 2012	31 July 2016	US
4	Univ of Massachusetts Med Sch Worcester	Immunity and virus disease	397,961	13,530,674	1 July 1980	31 March 2014	US
5	Cleveland Clinic Lerner Col/Med-Cwru	Antiviral actions of interferons	431,902	12,957,060	30 September 1985	31 January 2015	US
6	University of Virginia Charlottesville	Immune regulation of virus clearance and tissue injury at sites of infection	1,549,489	7,747,445	18 June 2009	31 May 2014	US
7	Dana-Farber Cancer Institute	Broad spectrum neutralizing human abs to SARS and related coronaviruses	1,083,666	5,418,330	9 June 2010	31 May 2015	US
8	New York Blood Center	Structure-based design of coronavirus subunit vaccines	911,083	4,555,415	21 May 2018	30 April 2023	US
9	Cleveland Clinic Lerner Col/Med-Cwru	Immune regulation of CNS viral recrudescence	375,123	4,501,476	15 May 2001	30 June 2012	US
10	New York Blood Center	Rational design and evaluation of novel mRNA vaccines against MERS-CoV	844,900	4,224,500	13 February 2018	31 January 2023	US

11	University of North Carolina Chapel Hill	System immunogenetics of SARS-CoV infection	739,947	3,699,735	5 August 2012	31 July 2017	US
12	Baylor College of Medicine	RBD recombinant protein-based SARS vaccine for biodefense	865,853	3,463,412	4 May 2012	30 April 2016	US
13	New York Blood Center	Enhancing potency of the MERS vaccine by a novel asp1alum adjuvant combination	690,074	3,450,370	1 August 2016	31 July 2021	US
14	Planet Biotechnology Inc	A MERS-CoV receptor decoy	600,081	2,400,324	1 June 2014	31 January 2018	US
15	University of Pennsylvania	Murine coronavirus neurovirulence role of type I interferon response	341,731	2,392,117	1 September 2012	31 May 2019	US
16	The Johns Hopkins University	Virus-like particle vaccine for SARS-CoV	386,641	2,319,846	15 April 2006	31 March 2012	US
17	Trudeau Institute Inc	Regulation of memory cd8 t cell recruitment to the lung	462,974	2,314,870	1 January 2008	31 December 2012	US
18	Cleveland Clinic Lerner Col/Med-Cwru	Regulation of b cells in the CNS	346,025	2,076,150	15 September 2013	30 June 2019	US
19	University of Iowa	Balancing protection versus immunopathology by RSV-specific memory cd8 t cells	376,900	1,507,600	1 August 2017	31 July 2021	US
20	University of Illinois at Chicago	Characterization of therapeutic human monoclonal antibodies against SARS	473,173	1,419,519	12 September 2009	31 August 2012	US
21	University of Iowa	Role of anti-SARS-CoV t cell response in pathogenesis	266,377	1,331,885	1 June 2011	31 May 2016	US

22	Texas Tech University	Collaborative research modeling immune dynamics of RNA viruses in reservoir and nonreservoir species	349,841	1,049,523	15 September 2015	31 August 2018	US
23	Yale University	The effectiveness of vaccines in clinical practice	157,391	944,346	1 August 2006	31 July 2012	US
24	Dartmouth College	Structure function and antigenicity of coronavirus spike proteins	631,914	631,914	9 February 2017	31 December 2017	US
25	Planet Biotechnology Inc	A mucosally targeted MERS-CoV vaccine produced in plants	236,488	472,976	17 February 2017	31 March 2018	US
26	New York Blood Center	Critical neutralizing domain-based vaccines against new SARS like virus hCoVemc	232,909	465,818	15 December 2013	30 November 2015	US
27	Cornell University Ithaca	Development of a subunit vaccine for MERS-CoV and other emerging coronaviruses	196,146	392,292	6 June 2018	31 May 2020	US
28	The University of Pittsburgh	Preclinical evaluation of an adenoviral-based MERS-CoV vaccine	190,815	381,630	10 July 2015	30 June 2017	US
29	University of Maryland Baltimore	Inhibition of the innate immune response by the SARS coronavirus	108,000	324,000	1 July 2009	31 May 2012	US
30	University of Iowa	A novel strategy for developing a SARS-CoV vaccine	28,412	142,060	19 July 2011	30 June 2016	US
31	University of North Carolina Chapel Hill	Unlocking zika virus immune control and pathogenesis with the collaborative cross	135,110	135,110	1 August 2015	31 July 2016	US
32	Protein Sciences Corporation	Development of a coronavirus vaccine	13,416	134,160	26 September	30 November	US

					2003	2012	
33	Protein Sciences Corporation	Development of a recombinant SARS vaccine	10,718	107,180	22 May 2003	30 November 2012	US
34	University of Iowa	Heteroclitic and modified t cell epitopes in coronavirus encephalomyelitis	28,536	57,072	18 April 2011	17 April 2013	US
35	The University of Tokyo	Nlrp3 inflammasome control of by viral infection	111,800	223,600	1 April 2015	31 March 2017	Japan
36	National Institute of Infectious Diseases	The development of a new vaccine against human coronavirus that causes severe pneumonia	48,100	144,300	1 April 2016	31 March 2019	Japan
37	National Institute of Infectious Diseases	Analysis of effect of changes in cell entry pathway of coronavirus on innate immune response	46,800	140,400	1 April 2017	31 March 2020	Japan
38	National Institute of Infectious Diseases	Study for side effects of inactivated SARS-CoV vaccine.	44,563	133,689	1 January 2010	31 December 2012	Japan

Table S3. Platform for detection and point-of-care diagnostics (Sub-cluster 1-3).

NO	Organization	Title	Estimated Average Cost/Fiscal Year (USD)	Estimated Total Cost (USD)	Start date	End date	Geographic Region
1	Medical College of Wisconsin	Rapid point of care diagnostic for bioterrorism	1,526,585	9,159,510	1 September 2006	31 August 2012	US
2	Manipal Academy of Higher Education	Gh13003 hospital-based surveillance of acute febrile illness in western GHAT region of	1,557,522	7,787,610	30 September 2013	29 September 2018	US
3	Columbia University Health Sciences	Diagnostic and prognostic biomarkers for viral severe lung disease	1,131,261	5,656,305	7 March 2014	28 February 2019	US
4	Laval University	Point of care microfluidic diagnosis system for rapid and sensitive detection of	811,888	4,059,440	1 July 2010	30 June 2015	US
5	Medical College of Wisconsin	Multiple POC device for antigen molecular detection of influenza other viruses	456,807	2,740,842	8 July 2008	30 June 2014	US
6	University of California San Francisco	A rapid pan viral microarray diagnostic for category ac biodefense pathogens	888,279	2,664,837	9 August 2010	30 June 2013	US
7	Boston College	New genetic tools for comparative analysis of emerging viruses and virus-host molecular interactions in reservoir hosts versus spillover hosts	313,000	626,000	1 April 2018	30 September 2019	US
8	Akonni Biosystems Inc	Respiratory panel flow strips a point of care molecular diagnostic test	300,000	300,000	1 March 2012	28 February 2013	US
9	Crosslife Technologies, Inc.	SBIR phase I rapid instrument free nucleic acid test for pathogens and biothreats	224,929	224,929	1 June 2017	31 May 2018	US
10	Arizona State University-Tempe Campus	Isolating viral particles from whole blood	69,165	207,495	7 February	31 January	US

					2012	2015	
11	University of Virginia Charlottesville	Yeast based assays for chemical screens against SARS-CoV targets	38,500	154,000	15 June 2008	28 February 2012	US
12	Tulane University of Louisiana	Epithelial cells lining salivary gland ducts are early target of SARS-CoV	37,186	37,186	1 May 2011	30 April 2012	US
13	office of Internatl Science &Engineering	Eapsi rapid smartphone-based paper assay fabrication for sensitive and specific detection of middle east respiratory syndrome coronavirus MERS-CoV	5,400	5,400	1 June 2016	31 May 2017	US
14	Kyoto Prefectural University of Medicine	Metagenomic research of virus infections in human clinical specimens by high-throughput DNA sequencing in Thailand	187,200	561,600	1 April 2011	31 March 2014	Japan
15	Nihon University	Establishment of gene diagnosis of feline infectious peritonitis by the analysis of the viral genome	44,200	132,600	1 April 2015	31 March 2018	Japan
16	Erasmus Universitair Medisch Centrum Rotterdam	Early detection of emerging viruses by next generation in situ hybridization	102,946	205,891	1 July 2014	30 June 2016	EU

Table S4. Structure-activity relationship modeling based virus prediction and activity modulation (Sub-cluster 2-1).

NO	Organization	Title	Estimated Average Cost/Fiscal Year (USD)	Estimated Total Cost (USD)	Start date	End date	Geographic Region
1	Blood Systems Research Institute	Modeling viral entry and its inhibition using SARS-CoV	406,843	2,034,215	15 December 2007	30 November 2012	US
2	Harvard University	Structure and mechanism of programmed ribosomal frameshifting in SARS coronavirus	375,687	1,127,061	7 February 2013	31 January 2016	US
3	Osaka University	Elucidation of the establishment and the virulence factors of reverse genetic manipulation system of MERS coronavirus	48,100	144,300	1 April 2016	31 March 2019	Japan
4	The University of Tokyo	Using the data assimilation technology construction of multiscale infection propagation model and evaluation	19,000	57,000	1 January 2011	31 December 2013	Japan
5	Yamaguchi University	Analysis of coronavirus evolution mechanism for the purpose of appearance prediction emerging coronavirus	21,700	43,400	24 April 2015	31 March 2017	Japan
6	Katholieke Universiteit Leuven	Virus discovery and epidemic tracing from high throughput metagenomic sequencing	1,168,428	3,505,285	1 June 2015	31 May 2018	EU

Table S5. Studies on design of antiviral agents based on the structure and function of viral and human receptor proteins in Korea (Sub-cluster 2-2).

NO	Organization	Title	Estimated Average Cost/Fiscal Year (USD)	Estimated Total Cost (USD)	Start date	End date	Geograp hic Region
1	University of Alabama at Birmingham	Antiviral drug discovery and development center overall	6,948,532	34,742,660	1 March 2014	28 February 2019	US
2	University of North Carolina Chapel Hill	Broad spectrum antiviral GS-5734 to treat MERS-CoV and related emerging CoV	1,310,955	6,554,775	9 August 2017	31 July 2022	US
3	University of Alabama at Birmingham	Inhibitors of coronavirus fidelity and cap methylation as broadly applicable	996,397	4,981,985	1 March 2014	28 February 2019	US
4	Phelix Therapeutics, LLC	Cathepsin l inhibitors as pan coronavirus therapeutics	1,460,695	4,382,085	1 March 2015	28 February 2018	US
5	Kansas State University	Small molecule protease inhibitors against MERS-CoV	775,916	3,879,580	15 May 2018	30 April 2023	US
6	The Johns Hopkins University	Applying human factors and mathematical modeling approaches to prevent transmission of high consequence pathogens	1,222,700	3,668,100	30 September 2015	29 September 2018	US
7	University of Minnesota Twin Cities	Receptor recognition and cell entry of coronaviruses	457,831	2,289,155	7 June 2016	31 May 2021	US
8	Emory University	Nox1 and nox2 as therapeutic targets in influenza	359,366	2,156,196	1 July 2012	30 June 2018	US
9	University of Minnesota Twin Cities	Receptor recognition mechanisms of coronaviruses	369,995	1,849,975	15 May 2010	30 April 2015	US
10	University of North Carolina Chapel Hill	Platforms for synthesis and testing of emerging and zoonotic viruses	614,822	1,844,466	1 March 2011	28 February 2014	US
11	University of Alabama at Birmingham	Novel nucleic acid binding proteins in emerging viruses	312,952	1,564,760	1 August 2016	31 May 2021	US

12	University of Illinois at Chicago	Development of plpro and 3clpro protease inhibitors as novel SARS therapeutics	758,511	1,517,021	26 July 2010	25 July 2012	US
13	Kineta Inc	Development of novel isoflavone drugs as broad-spectrum antivirals	752,081	1,504,162	22 June 2016	31 May 2018	US
14	Blood Systems Research Institute	Broad spectrum antivirals targeting envelope proteolysis and viral uncoating	249,518	748,554	1 June 2013	31 May 2016	US
15	Drexel University	Coronavirus modulation of cellular microRNAs in the liver role in hepatitis	212,438	637,314	29 August 2011	31 July 2014	US
16	Cornell University Ithaca	Spike protein cleavage activation and emergence of coronaviruses with extended tr	209,452	628,356	15 August 2014	31 July 2017	US
17	Cornell University Ithaca	Priming and fusion activation of the SARS coronavirus spike glycoprotein	190,575	571,725	25 June 2009	31 May 2012	US
18	New York Blood Center	A novel nanobody with good druggability to prevent and treat MERS-CoV infection	241,450	482,900	8 December 2016	30 November 2018	US
19	Autoimmune Technologies LLC	Peptide inhibitors of coronavirus entry	225,000	450,000	15 June 2014	31 May 2016	US
20	Glsynthesis Inc	Solid state synthesis and applications of oligophenyleneethynes	191,458	382,916	1 July 2010	30 June 2012	US
21	University of Maryland Baltimore	Repurposing of abl kinase inhibitors for treatment of coronavirus infections	41,673	125,019	1 February 2015	31 January 2018	US
22	Vanderbilt University	Coronavirus antiviral nucleoside analogs inhibition and reduced susceptibility	28,966	86,898	30 September 2017	29 September 2020	US
23	Dynport Vaccine Company, Llc	Phase I clinical trial unit for therapeutics phase I trial for MERS-CoV	59,564	59,564	19 August 2016	5 December 2016	US

24	Vanderbilt University	Viral protein determinants of coronavirus replication fidelity	49,214	49,214	1 September 2013	31 August 2014	US
25	Tokyo University of Pharmacy and Life Science	Integrated medicinal chemistry research of intractable diseases based on peptidic small molecules	182,000	728,000	1 April 2011	31 March 2015	Japan
26	Kyoto Pharmaceutical University	Design of nonpeptide inhibitors based on structure analyses of the protease inhibitor complex	52,000	156,000	1 April 2013	31 March 2016	Japan
27	Nippon Veterinary and Life Science University	The development of specific anti-coronavirus drugs using the novel glycosidase inhibitors	48,100	144,300	1 April 2016	31 March 2019	Japan
28	Nippon Veterinary and Life Science University	Development of endosome targeted peptide inhibitors against enveloped viruses via endosomal pathway.	44,200	88,400	1 January 2011	31 December 2012	Japan
29	Tokyo University of Pharmacy and Life Science	Study on medicinal chemistry of reversible cysteine protease inhibitors for the treatment of infectious diseases	37,700	75,400	1 January 2011	31 December 2012	Japan
30	Tokyo University of Pharmacy and Life Science	Development of nonpeptide type SARS virus protease inhibitors with aryl ketone structure	16,000	32,000	1 January 2011	31 December 2012	Japan
31	University of Bristol	Synthesis of smart virus like hierarchical structures based on polymer peptide conjugates and the potential application in drug delivery	122,285	244,569	1 July 2012	30 June 2014	EU

Table S6. Infectious Disease Epidemiological Investigation & Animal and Environmental Ecology (Cluster 3).

NO	Organization	Title	Estimated Average Cost/Fiscal Year (USD)	Estimated Total Cost (USD)	Start date	End date	Geographic Region
1	Leidos Biomedical Research, Inc.	IGF::OT::IGF operation of government-owned contractor-operated (GOCO) R&D facilities	9,820,006	49,100,031	26 September 2015	25 September 2020	US
2	Leidos Biomedical Research, Inc.	Respiratory viruses research	4,662,028	23,310,140	26 September 2015	25 September 2020	US
3	Leidos Biomedical Research, Inc.	Emerging/re-emerging and related respiratory diseases NIAID/DCR	2,256,233	22,562,327	26 September 2008	25 September 2018	US
4	George Washington University	Ecology of MERS-CoV in camels humans and wildlife in Ethiopia	2,487,071	9,948,284	1 September 2018	31 August 2022	US
5	Montana State University - Bozeman	Dynamics of zoonotic systems human bat pathogen interactions	1,650,000	6,600,000	1 September 2017	31 August 2021	US
6	Dynport Vaccine Company, Llc	Phase i clinical trial unit for therapeutics against infectious diseases evaluation of therapeutics for MERS-CoV	857,452	2,572,356	23 August 2016	28 June 2019	US
7	Ecohealth Alliance Inc	Risk of viral emergence from bats	503,136	2,515,680	18 September 2008	31 August 2013	US
8	Wadsworth Center	Emerging infectious diseases training program	222,675	2,449,425	1 July 2004	31 July 2015	US
9	Harvard School of Public Health	Epidemiology of infectious diseases and biodefense	135,759	2,172,144	30 September 1998	31 May 2014	US
10	Fred Hutchinson Cancer Research Center	Realtime tracking of virus evolution for vaccine strain selection and epidemiological investigation	409,400	2,047,000	23 August	31 May 2021	US

					2016		
11	University of Michigan at Ann Arbor	Career wastewater treatment as a conduit and control of emerging respiratory viruses in the environment	400,000	2,000,000	1 May 2014	30 April 2019	US
12	University of Utah	Epidemiology and etiology of hospitalized pneumonia in children	555,082	1,665,246	1 September 2009	31 August 2012	US
13	North Carolina State University Raleigh	Career big computation and the management of emerging infectious diseases	180,310	901,550	1 June 2016	31 May 2021	US
14	University of Colorado Denver	Epidemiology and pathogenesis of novel coronaviruses	132,300	793,800	1 May 2008	30 April 2014	US
15	Pasteur Institute from Madagascar	Investigating seasonal drivers of viral zoonoses from Madagascar fruit bats	134,900	674,500	2 May 2017	30 April 2022	US
16	University of California Davis	Epidemiology of zoonotic viruses in forest communities in a key biodiversity area of rural myanmar	121,699	608,495	15 June 2016	31 May 2021	US
17	Arizona State University-Tempe Campus	Iii small data management for real time data driven epidemic spread simulations	157,296	471,888	1 September 2013	31 August 2016	US
18	University of Maryland Baltimore	Diabetic comorbidity and MERS coronavirus pathogenesis	214,359	428,718	13 June 2016	31 May 2018	US
19	National Academy of Sciences	Forum on microbial threats	60,000	300,000	12 September 2014	31 August 2019	US
20	University of Maryland Baltimore	The role of diabetes in middle east respiratory syndrome coronavirus pathogenesis	58,282	116,564	1 August 2018	31 July 2020	US
21	Colorado State University-Fort Collins	2nd international infectious diseases of bats symposium	6,000	6,000	1 May 2017	30 April 2018	US
22	Nagoya University	Appearance prediction of fruit bats from emerging infectious diseases in southeast Asia and Oceania	172,900	864,500	1 April 2013	31 March 2018	Japan
23	National Institute of Infectious Diseases	Survey of middle east respiratory syndrome coronavirus of the dromedary in Ethiopia	137,800	413,400	1 April 2017	31 March	Japan

						2020	
24	Erasmus Universitair Medisch Centrum Rotterdam	Anticipating the global onset of novel epidemics	3,670,585	18,352,925	1 November 2011	31 October 2016	EU
25	Victoriano Martinez Riomansa Sl	A novel filtration technology targeted at enhancing the European healthcare systems efforts in restraining the spread of the hospital infection	749,257	1,498,514	1 January 2011	31 December 2012	EU

Table S7. Researches related to mechanisms of infection, the life cycle of SARS-CoV-2, and identification of virus-host interaction mechanism in Korea (Sub-cluster 1-1).

NO	Organization	Title	Estimated Average Cost/Fiscal Year (USD)	Estimated Total Cost (USD)	Start date	End date
1	National Institutes of Health	Severe acute respiratory syndrome (SARS) pathogenesis and treatment method development using a porcine coronavirus infection model	24,545	24,545	1 July 2005	1 July 2006
2	Kookmin University	A study on the enzymatic characterization of virus helix enzyme in severe acute respiratory syndrome	31,818	31,818	1 December 2005	1 November 2006
3	Kookmin University	Reaction mechanism study on SARS Coronavirus helicase and its applications to develop the inhibitors	45,736	137,209	1 November 2013	31 October 2016
4	Kangwon National University	Database construction and collection of zoonotic pathogens in Korea	89,091	445,455	1 July 2010	31 July 2015
5	Kyungpook National University	Functional studies on roles of MAPK signaling pathways in porcine coronavirus replication	37,030	111,091	1 September 2012	31 August 2015
6	Chonbuk National University	A study for zoonotic potential of Staphylococcus spp. in wildlife ecosystem: anti-microgram and molecular characterization	44,093	132,279	1 November 2016	31 October 2019
7	Kyungpook National University	Functional studies on roles of intracellular signaling pathways in novel porcine delta coronavirus replication	30,704	92,111	1 November 2015	31 October 2018
8	Chonbuk National University	A genetic characteristic study for zoonotic potential of several pathogens from bat in Korea: Molecular characterization and identification of zoonotic viruses and parasites from bat using next genera...	15,152	45,455	1 June 2018	31 May 2021
9	Kyungpook National University	Investigation of virulence factors for porcine epidemic diarrhea virus and development of reverse genetics-based attenuated vaccine platform	6,818	34,091	1 June 2018	31 May 2023
10	Kookmin University	Reaction mechanism study on MERS Coronavirus helicase in the process of viral genome replication	30,303	90,909	1 March 2020	28 February 2023

11	Hallym University	Study on mechanisms of hepatitis C virus replication using host cellular proteins	181,818	363,636	1 June 2016	31 May 2018
12	Gyeongsang National University	Development of antiviral biomaterial based on the glycosidase and protease	34,545	172,727	1 May 2009	31 March 2014
13	Kookmin University	Mechanistic study on SARS Coronavirus helicase in the process of viral genome replication	18,272	54,816	1 September 2010	31 August 2013
14	Chosun University	Study on interaction between virus and membrane microdomains using model systems	9,091	45,455	1 June 2018	31 May 2023
15	Chungnam National University	Identification of mechanism of coronavirus cross-species transmission and pathogenesis mediated by host proteases	45,455	136,364	1 March 2020	28 February 2023
16	Chungnam National University	Studies on the mechanism of the emergency of mutant coronaviruses with the deletion in the spike protein and its application	26,515	79,545	1 June 2017	31 May 2020
17	Chonbuk National University	Genetic and Biological characterization of important fungi from bats in Korea	11,364	34,091	1 June 2019	31 May 2022
18	Kyungpook National University	Functional characterization of the interaction between the porcine epidemic diarrhea virus (PEDV) spike (S) protein and its cellular receptor	25,909	51,818	1 May 2009	30 April 2011
19	Chonbuk National University	Investigation on the infection mechanism and the control of RNA viruses through monoclonal antibody	6,363	31,813	1 September 2010	31 August 2015
20	Kookmin University	Mechanistic studies on the helicase of MERS coronavirus	60,606	181,818	1 March 2017	29 February 2020
21	Hallym University	Metagenomic study and molecular screening of rodent (Apodemus agrarius)-borne biothreat viruses using next-generation sequencing and targeted RT-PCR	34,091	68,182	1 June 2019	31 May 2021
22	Dankook University	Influence of heavy metal in the ultrafine particulate matter on the respiratory virus infection and respiratory inflammation	20,273	60,818	1 June 2019	31 May 2022
23	Gunsan University Industry-University Cooperation Foundation	Investigation of the substrate recognition mechanism and development of natural inhibitors of SARS 3CL protease	15,152	45,455	1 May 2010	30 April 2013

24	Hallym University	Development of Spike protein expression technology for each genotype of swine pandemic virus	61,364	61,364	1 June 2011	31 May 2012
25	Hallym University	Development of Spike gene recombination protein expression technology for porcine coronavirus diagnosis	57,273	57,273	1 June 2010	31 May 2011
26	Seoul National University Hospital	A study on viral shedding of MERS-CoV	81,818	163,636	1 November 2015	31 October 2017
27	Kyunghee University	Development of transgenic mouse models for middle east respiratory syndrome coronavirus infection	34,091	136,364	3 December 2015	30 November 2019
28	Chungnam National University Hospital	Virus and Antibody kinetics of MERS-CoV confirmed cases	40,909	81,818	1 November 2015	31 October 2017
29	Kookmin University	Screening of RNA aptamers that specifically bind to Nucleocapsid and Spike proteins of SARS Coronavirus and inhibit their activities	25,000	50,000	1 May 2009	31 March 2011
30	Chungbuk National University	Phylogenetic study of SARS-coronavirus and new virus	102,727	102,727	1 June 2003	1 May 2004
31	Hallym University	Researches on Viral Infectious Diseases	10,707	96,364	1 June 2005	1 February 2014
32	Center for Disease Control	Immunogenocity of SARS-CoV receptor binding protein expressed on coxsackie viral vector	30,455	60,909	1 January 2010	31 December 2011
33	WizTul BioLab	Improved high-efficiency T4 DNA ligase	60,636	60,636	1 June 2016	28 February 2017
34	Kyungpook National University	Molecular characterization and diagnostic tool development of porcine delta coronavirus in Korea	54,735	109,471	1 January 2016	31 December 2017
35	Animal and Plant Quarantine Agency	Genetic analysis of swine pandemic virus	8,727	43,636	1 August 2003	1 December 2007
36	Animal and Plant Quarantine Agency	Porcine virus diarrhea hospital human investigation	2,727	13,636	1 June 2003	1 December 2007
37	Animal and Plant Quarantine Agency	Animal pathogenic virus genome construction research	30,303	90,909	1 January	31 December

	2008	2010

Table S8. Researches related to platform for immunological response to viral infection and for vaccine development in Korea (Sub-cluster 1-2).

NO	Organization	Title	Estimated Average Cost/Fiscal Year (USD)	Estimated Total Cost (USD)	Start date	End date
1	Korea Research Institute of Chemical Technology	Development of convergence solution for new virus infection	13,167,521	26,335,042	1 August 2016	31 December 2017
2	Korea Institute of Oriental Medicine	Convergent solution for emerging virus infection	303,030	909,091	1 August 2016	31 July 2019
3	Korea University	Development of next-generation universal vaccine to control viral infectious diseases	181,818	181,818	1 December 2015	30 November 2016
4	Seoul National University	Development of therapeutic antibodies against Middle East respiratory syndrome coronavirus and Zika virus	99,273	496,364	1 July 2016	31 March 2021
5	Seoul National University	The Interferon-targeting therapeutic mechanism to overcome the pandemic respiratory viral infection	20,833	125,000	1 June 2016	31 May 2022
6	Seoul National University	Identification of TFH differentiation and target AGS for MERS-CoV vaccine using virus-like particles	90,909	272,727	1 March 2017	29 February 2020
7	Chonnam National University	Establishment of experimental models for evaluating the safety and efficacy of antiviral drugs	131,091	655,455	1 May 2009	31 March 2014
8	Celltrion Co., Ltd.	Development of Therapeutic Antibody for MERS-coronavirus	212,121	636,364	3 December 2015	30 November 2018
9	Catholic University	Development of Middle East Respiratory Syndrome Coronavirus (MERS-CoV) based on RNA vaccine platform	128,773	515,091	3 December 2015	30 November 2019

10	Korea Research Institute of Bioscience and Biotechnology	Development of Immunogen Candidates for the MERS Vaccine	110,795	443,182	3 December 2015	30 November 2019
11	Korea Central Academy	Development of animal model-based evaluation system for the MERS vaccine candidates	89,489	357,955	3 December 2015	30 November 2019
12	International Vaccine Research Institute	Discovery of MERS-CoV vaccine Candidate and Evaluation of Vaccine efficacy using in vitro system	79,545	318,182	3 December 2015	30 November 2019
13	Seoul National University	The verification of clinical efficacy and preventive effect for MERS-CoV mucosal vaccine candidate against respiratory viral infection	47,341	189,364	3 December 2015	30 November 2019
14	Korea University	Evaluation of neutralization of therapeutic antibody against MERS-coronavirus	45,455	136,364	3 December 2015	30 November 2018
15	National Medical Center	Immune response against MERS-Coronavirus infection in humans	40,909	81,818	1 November 2015	31 October 2017
16	Chonbuk National University	Development of experimental live attenuated MERS-CoV vaccines	119,318	477,273	3 December 2015	30 November 2019
17	Korea Research Institute of Bioscience and Biotechnology	Evaluation of safety and immunogenicity of Middle East Respiratory Syndrome Coronavirus (MERS-CoV) vaccine candidates in non-human primate	61,364	122,727	1 October 2018	30 November 2019
18	Konkuk University	MERS-CoV eS1-770 vaccine antigen construction and efficacy evaluation	118,182	472,727	17 December	30 November

					2015	2019
19	Seoul Asan Hospital	Prediction of weaning from veno-venous extracorporeal membrane oxygenation in adult patients with severe acute respiratory failure: Korean Exracorporeal Lung Support Organization (KELSO) score	59,091	118,182	11 May 2016	31 October 2017
20	Konyang University	Immune response to SARS coronavirus N antigen and vaccine candidate study	45,455	45,455	1 December 2005	1 December 2006
21	Center for Disease Control	Development of therapeutic monoclonal antibodies against MERS-CoV infection	332,727	998,182	1 January 2016	31 December 2018
22	Korea Research Institute of Chemical Technology	Development of human DPP4 expressing transgenic mouse	90,909	272,727	22 July 2016	31 December 2018
23	Yonsei University	Development of fluorescence expressing MERS-CoV for drug and vaccine evaluation	90,909	181,818	27 June 2016	31 December 2017
24	Korea Research Institute of Bioscience and Biotechnology	Purification of MERS-CoV antigen and Production of ELISA kit	123,966	123,966	20 June 2016	31 December 2016
25	Korea University	Construction of trimeric MERS-CoV Spike protein for screening of antibodies against MERS-CoV	72,727	72,727	1 November 2016	31 August 2017
26	Osong Medical Innovation Foundation	Screening of high affinity MERS-CoV monoclonal antibodies using phage display library	54,545	54,545	24 October 2016	23 December 2016
27	Konyang University	SARS coronavirus nucleocapsid DNA vaccine development	60,000	60,000	1 July 2006	1 April 2007
28	Center for Disease Control	Development and evaluation of an avirulent MERS-CoV	121,212	363,636	1 January	31

		for vaccine candidate			2019	December 2021
29	Center for Disease Control	Validation of MERS-CoV broadly neutralizing antibodies in mouse in vivo for practical use	121,212	363,636	1 January 2019	31 December 2021
30	Korea University	Construction of respiratory viral immunogenic protein probes for screening of neutralizing antibodies	36,364	72,727	6 February 2017	31 December 2018
31	Center for Disease Control	Expression and immunogenicity study of Coxsackievirus vector of SARS-CoV receptor binding protein	28,788	86,364	1 January 2008	31 December 2010
32	Korea Technology Exchange	Development of VSF for the treatment of viral diseases	17,273	17,273	1 May 2003	1 December 2003
33	Catholic University	Development of standard protocol for measuring the amount of vaccine candidate and the titer of antibody for MERS-CoV (1)	154,545	309,091	16 February 2016	30 November 2017
34	Sejong University	Evaluation of the in vitro and in vivo efficacy of adjuvants for MERS-vaccine (1)	90,909	181,818	16 February 2016	30 November 2017
35	Chonbuk National University	Development of standard protocol for measuring the amount of vaccine candidate and the titer of antibody for MERS-CoV (2)	22,727	45,455	16 February 2016	30 November 2017
36	National Institute of Food and Drug Safety Evaluation	Study on the establishment of testing method for antibody titer and reference sera for evaluation of virus vaccine	290,909	581,818	1 January 2019	31 December 2020

37	Agriculture, Forestry and Livestock Quarantine Headquarters	Development of killed vaccine against PEDV, PDCoV and Rotavirus in pig	5,779	11,557	1 January 2018	31 December 2019
38	Korea B & P Co., Ltd.	Development and industrialization of live attenuated bovine rotaviruses and coronavirus multivalent vaccines matched currently circulating viruses in South Korea	37,576	112,727	10 April 2009	9 April 2012
39	Wells Bio Co., Ltd.	Development of a high sensitivity and high specificity immunochromatographic test kit for rapid detection of MERS CoV antigen	245,455	245,455	15 May 2019	14 May 2020

Table S9. Platform for detection and point-of-care diagnostics in Korea (Sub-cluster 1-3).

NO	Organization	Title	Estimated Average Cost/Fiscal Year (USD)	Estimated Total Cost (USD)	Start date	End date
1	Kookmin University	Severe Acute Respiratory Syndrome Coronavirus Heliase Reactor Study and Activity Inhibition RNA Aptamers	89,091	89,091	1 November 2006	1 October 2007
2	Kookmin University	SiNW FET-based Bio-Molecular Multiplexed Diagnosis Systems	60,982	182,945	1 November 2013	31 October 2016
3	Yonsei University	Development of organic-inorganic hybrid 3D nanostructure	3,385	416,364		31 August 2022
4	Konkuk University	Detection and control of airborne MERS virus bioaerosols via airborne fine biomaterial control technology	30,172	90,515	1 November 2015	31 October 2018
5	Sogang University	Visualization of influenza virus using Qdot-aptamer beacon and 3D photonic crystals for enhancement of fluorescent signal	45,455	45,455	1 November 2016	31 October 2017
6	Korea Advanced Institute of Science and Technology	Development of rapid immunoassay and monitoring systems for zoonosis	17,636	88,182	16 August 2010	31 July 2015
7	Seoul Asan Hospital	Advancement of Specimen Processing and Evaluation of Ultrasensitive Diagnostic Platform Technologies for Emerging Viruses	30,636	153,182	1 July 2016	31 March 2021
8	Chonbuk National University	The development of prompt and customized genetic engineering technology-based approaches for the control of disastrous infectious diseases	53,036	477,328	1 June 2017	28 February 2026
9	Konkuk University	Development of platform technology for highly sensitive detection of emerging viruses using gene amplification and fluorescence monitoring	48,182	240,909	1 July 2016	31 March 2021
10	Sungkyunkwan University	Biomimetic LSPR nanosensor for rapid diagnosis of lethal epidemic virus diseases	11,364	34,091	1 June 2018	31 May 2021
11	Korea Institute of Science and Technology	Highly multiplexed particle-qPCR for respiratory viral infection	90,909	454,545	1 July 2016	31 March

						2021
12	Samsung Seoul Hospital	Development of genetic-based point-of-care multiplexed diagnostic system	37,273	186,364	1 July 2016	31 March 2021
13	Gwangju Institute of Science and Technology	The development of monitoring system for detecting of transmitted disease in the inside environments	90,909	90,909	27 March 2018	26 September 2018
14	Kwangwoon University	Ultrasensitive MERS-CoV Biosensor Composed of Multi-functional DNA 3 Way-Junction Probe based on Electrochemical-Surface Enhanced Raman Spectroscopy (EC-SERS) Dual Detection System	15,152	45,455	1 June 2018	31 May 2021
15	Korea Research Institute of Bioscience and Biotechnology	BT / IT / NT convergence technology for biorisk field diagnosis and monitoring	45,455	45,455	1 August 2008	31 December 2008
16	Korea Research Institute of Bioscience and Biotechnology	Next-generation virus detection and control technology development	17,455	87,273	1 January 2019	31 December 2023
17	Geneclone Corporation	Development of rapid diagnosis reagent and protein vaccine production technology for intermediate and intermediate respiratory syndrome (SARS)	79,091	79,091	1 April 2004	1 March 2005
18	Genolution Inc.	Developing Multi-plex and portable nucleic acid extraction instrument and reagents on-site emergency test of infectious diseases	538,405	1,615,216	1 May 2016	31 December 2018
19	Korea Electronics Technology Institute	Development of portable biosensing system for the detection of acute and infectious viral diseases	623,727	2,494,909	1 December 2015	30 November 2019
20	Biometrics Technology Co., Ltd.	Development and production of diagnostics for rapid diagnosis of respiratory diseases	190,909	572,727	1 September 2014	31 August 2017

21	Center for Disease Control	Development of Strategy for Control of Emerging and Re-Emerging Viruses	454,545	454,545	1 December 2004	1 December 2005
22	Catholic University	Development of diagnostic algorithm and evaluation of rapid isothermal amplification assay	88,636	354,545	1 April 2016	31 December 2019
23	Konkuk University	Development of rapid diagnosis technology for multiple influenza virus strains using isothermal gene amplification	30,606	91,818	3 December 2015	30 November 2018
24	Seoul Asan Hospital	Diagnostic usefulness of newly developed diagnostic tests for emerging infectious diseases	45,455	136,364	3 December 2015	30 November 2018
25	Sogang University	Development of biosensor technology for detecting corona virus based on graphene oxide-gold nanoparticles	49,273	49,273	1 March 2017	31 December 2017
26	Center for Disease Control	Development of diagnostic resources for the rapid response of emerging respiratory viruses	181,818	545,455	1 January 2016	31 December 2018
27	Rapigen Inc.	Evaluation of MERS-CoV diagnosis kits for clinical application	272,588	272,588	24 December 2015	23 October 2016
28	Wonkwang University	Evaluation of MERS-CoV diagnosis kits for clinical application	263,636	263,636	24 December 2015	23 October 2016
29	Seoul National University Hospital	Evaluation of MERS-CoV diagnosis kits for clinical application	181,818	181,818	24 December	23 October 2016

					2015	
30	Kogene Co., Ltd.	Evaluation of MERS-CoV diagnosis kits for clinical application	170,909	170,909	29 December 2015	28 October 2016
31	Center for Disease Control	Improvement of genetic diagnostic system and development of serological analysis for MERS-CoV detection	51,515	154,545	1 July 2014	31 December 2016
32	Center for Disease Control	A study on the construction of a dual antigen / antibody detection ELISA diagnostic system using SARS (severe acute respiratory syndrome) recombinant protein	17,576	52,727	1 January 2005	31 December 2007
33	Yonsei University	Development of new technology for diagnosis of SARS virus using Aptamer	125,455	125,455	18 September 2007	17 June 2008
34	PCL Co., Ltd.	Development of rapid diagnostic system for detection of Human coronaviruses based on POCT	90,909	90,909	7 July 2017	6 July 2018
35	Flexense Co., Ltd.	Development of nano-pattern plastic sensor based on surface plasmon for diagnosis of viral diseases	195,602	391,205	14 December 2016	13 December 2018
36	Paxgenbio Co., Ltd.	Development of Universal Lateral flow assay system and STI10 kit	181,818	181,818	20 November 2015	19 November 2016
37	Paxgenbio Co., Ltd.	Developed molecular diagnostic kit for influenza A, Influenza B, RSV field diagnosis based on isothermal amplification and lateral flow analysis technology	102,727	102,727	1 October 2019	30 September 2020
38	Hanyang University	Development of rapid molecular diagnosis kit for companion animals using real-time PCR method	79,871	79,871	1 May 2016	30 April 2017

39	JS Biotech	Development of simultaneous detection technology of coronavirus using on-site diagnostic system	56,909	56,909	1 December 2016	30 November 2017
40	Yonsei University	Development of Safety and Efficacy Evaluation Technology for MERS In Vitro Diagnostics	138,273	138,273	1 June 2016	28 February 2017
41	National Institute of Food and Drug Safety Evaluation	Research and development of guidelines for performance evaluation of diagnostic biosensor medical device appropriate for POCT environment (1)	181,818	181,818	1 January 2016	31 December 2016
42	Sungshin Women's University	Study on production of certified reference materials (CRM) for molecular diagnostic of infectious diseases (1)	33,476	66,953	16 February 2016	30 November 2017
43	Seoul National University	Development of diagnostic and evaluation techniques using n-protein of SARS (Severe acute respiratory syndrome virus)	63,636	63,636	1 March 2006	1 November 2006
44	Ministry of Agriculture, Food and Rural Affairs, Ministry of Agriculture, Food and Rural Affairs, Animal and Plant Hygiene Research Division	Establishment of genetic diagnostic method for the detection of Middle East Respiratory Syndrome virus	25,636	25,636	1 January 2014	31 December 2014
45	Animal and Plant Quarantine Agency	Domestic separation SARS corona, Rotavirus characterization and diagnosis method development	236,364	472,727	1 January 2005	1 December 2006

Table S10. Structure-activity relationship modeling based virus prediction and activity modulation in Korea (Sub-cluster 2-1).

NO	Organization	Title	Estimated Average Cost/Fiscal Year (USD)	Estimated Total Cost (USD)	Start date	End date
1	Yonsei University	Basic research on new drug candidate discovery based on cheminformatics	296,970	890,909	1 September 2006	31 August 2009
2	Korea Research Institute of Bioscience and Biotechnology	Development of viral recombination prediction and validation technique using bioinformatics	30,303	272,727	1 January 2014	31 August 2022
3	Kingdom University	Big-data analysis on viral infection using epigenetic information	48,158	144,475	1 June 2016	31 May 2019
4	Seoul National University	Simulation study for the prediction of zoonotic infection risk using genetic variation markers of viruses	26,515	79,545	1 June 2017	31 May 2020
5	Korea University	Development of universal countermeasures against viral diseases based on X-ray free electron laser technology	140,151	560,605	1 December 2016	30 November 2020
6	Seoul National University	Research on genetic variations associated with local adaptation of indigenous African cattle via modeling large-scale SNP sequence data using deep learning	31,818	63,636	1 November 2016	31 October 2018
7	Center for Disease Control	Variation predict and viral attenuation of MERS-CoV	254,545	763,636	1 January 2016	31 December 2018

Table S11. Studies on design of antiviral agents based on the structure and function of viral and human receptor proteins in Korea (Sub-cluster 2-2).

NO	Organization	Title	Estimated Average Cost/Fiscal Year (USD)	Estimated Total Cost (USD)	Start date	End date
1	Sungkyunkwan University	Discovering ligands that bind to RNA-like knot structures through computerized compound group searches and biochemical activity analysis	22,727	45,455	1 September 2003	1 August 2005
2	Sungkyunkwan University	RNA Pseudoknot Targeted Virtual Screening: Discovery of Novel Ligands that Regulate -1 Ribosomal Frameshifting	6,364	6,364	1 November 2005	1 October 2006
3	Sungkyunkwan University	Development of Candidate Anti-frameshift Drugs against SARS Coronavirus through Lead Optimization	104,545	104,545	1 July 2008	30 June 2009
4	Pusan National University	Studies on development of novel antiviral agent based on mechanism of target enzyme, S-adenosylhomocysteine hydrolase (SAH)	22,727	45,455	1 May 2005	1 March 2007
5	Institut Pasteur Korea	Preparedness to Emerging Viruses	500,035	3,000,210	30 June 2017	31 December 2022
6	Ilyang Pharmaceutical Co., Ltd.	Deriving new candidates for development of therapeutics in the Middle East Respiratory Syndrome	152,727	763,636	1 July 2016	31 March 2021
7	Ulsan University	Safe and high-throughput pseudo virus-based screening of natural compounds to inhibit emerging viruses	26,617	79,852	1 June 2017	31 May 2020
8	Ewha Women University	Development of antivirus agents targeting active sites of MERS-CoV proteases	22,815	45,630	1 November 2016	31 October 2018
9	Hallym University	Development of therapeutic target and candidate for immunotherapy against middle east respiratory syndrome coronavirus (MERS-CoV)	83,636	418,182	1 July 2016	31 March 2021
10	Konkuk University	Structural Study of SARS Helicase and its Complex with RNA Aptamers	14,300	42,900	1 May 2009	30 April 2012
11	Sungkyunkwan University	Development of antiviral drugs that control new infectious	20,909	20,909	1 November	31 October

		diseases through leading material optimization research			2009	2010
12	Korea Advanced Institute of Science and Technology	Development of novel therapeutics for infectious diseases through activation of innate immune system	178,182	356,364	1 November 2015	31 October 2017
13	Korea Research Institute of Bioscience and Biotechnology	Development of biomaterial for virus infection control	287,091	1,435,455	1 May 2009	31 March 2014
14	Korea Research Institute of Bioscience and Biotechnology	Optimization and action mechanism study of biomaterial (JSC, KW-200) for virus infection control	103,636	518,182	1 May 2009	31 March 2014
15	Korea Research Institute of Bioscience and Biotechnology	Development of eco-friendly active biomaterials for preventing and treating infection inflammation	222,197	2,666,364	1 January 2009	31 December 2020
16	Korea Research Institute of Bioscience and Biotechnology	Development of candidates for treatment for infectious viral infections	45,455	45,455	1 January 2008	31 December 2008
17	Jeollabuk-do Biological Industry Promotion Agency	Antiviral and anti-harmful microorganism preparations using natural plant extracts	7,891	7,891	1 March 2012	28 February 2013
18	Chungnam National University	Development of feline infectious peritonitis antiviral agent	108,943	108,943	1 June 2014	31 May 2015
19	Immunemed Co., Ltd.	Identification of new cytokine VSF function and production of recombinant VSF secretion strain	80,909	80,909	1 December 2005	1 November 2006
20	Wonkwang University	Common infectious disease treatment research and development project	236,364	1,181,818	1 May 2005	1 April 2010
21	Chonbuk National University	Development of recombinant subunit vaccine candidates for the protection against MERS-CoV infection	68,182	272,727	3 December 2015	30 November 2019
22	Korea Advanced Institute of Science and Technology	Protein engineering-based MERS virus infection suppression protein development and MERS virus surface protein structure identification	9,091	9,091	1 April 2017	31 December

						2017
23	Gueulri Co., Ltd.	Development of natural product-derived agents for the prevention and response to virus infection in animals	157,091	157,091	1 July 2013	30 June 2014
24	Gachon University Industry-University Cooperation Foundation	Development and industrialization of treatment materials for chronically infected Hefei virus using domestic plant resources	34,545	172,727	26 December 2011	25 December 2016
25	Immunemed Co., Ltd.	Research on the development of therapeutic agents for viral diseases through the discovery of new antiviral substances and production of recombinant proteins	30,000	90,000	1 May 2006	30 April 2009

Table S12. Infectious Disease Epidemiological Investigation & Animal and Environmental Ecology in Korea (Cluster 3).

NO	Organization	Title	Estimated Average Cost/Fiscal Year (USD)	Estimated Total Cost (USD)	Start date	End date
1	Seoul National University	Research on basic technology for prevention of common infectious diseases	301,515	904,545	1 September 2006	1 August 2009
2	Korea University	IoT Based Realtime Tracking and Management of Infectious Diseases in Hospital	90,895	90,895	1 December 2015	30 November 2016
3	Seoul City University	Studies on the Development of MERS Diffusion Route Detection and Prevention Technology: focus on public transportation users	45,455	227,273	29 November 2019	28 November 2024
4	Inha University	Mathematical Control Strategies for Effective Preventive Measures Against Epidemics: Macroscopic and Microscopic Viewpoints	26,515	79,545	1 June 2017	31 May 2020
5	Konkuk University	Mathematical models of contact management and spread of infectious diseases	90,909	181,818	7 November 2018	31 December 2019
6	Seoul National University Hospital	Research on the pursuit of immunocompromised MERS	363,636	363,636	24 December 2015	23 October 2016
7	National Medical Center	Clinical and immunological study of 3 years after infection in MERS patients	227,271	227,271	8 December 2017	7 December 2018
8	National Medical Center	Clinical and immunological study of 4 years after infection in MERS patients	181,816	181,816	1 February 2019	31 December 2019

9	Woori Airtech Korea Co., Ltd.	Developed mobile sound pressure booths to prevent secondary infections and medical staff when treating various infectious diseases such as MERS	58,237	58,237	1 June 2016	28 February 2017
10	National Institute of Animal Science	Analysis and monitoring of clinical and epidemiological features of African Swine Fever	45,455	90,909	1 March 2020	31 December 2021
11	Agriculture, Forestry and Livestock Quarantine Headquarters	Studies on surveillance for porcine delta coronavirus in Korea and its pathogenicity	39,455	78,909	1 January 2016	31 December 2017
12	Agriculture, Forestry and Livestock Quarantine Headquarters	Monitoring and biological characteristics of canine parvovirus2 and feline coronavirus in Korea	34,802	104,405	1 January 2019	31 December 2021
